# PKC Signaling Regulates Drug Resistance of the Fungal Pathogen *Candida albicans* via Circuitry Comprised of Mkc1, Calcineurin, and Hsp90

**DOI:** 10.1371/journal.ppat.1001069

**Published:** 2010-08-26

**Authors:** Shantelle L. LaFayette, Cathy Collins, Aimee K. Zaas, Wiley A. Schell, Marisol Betancourt-Quiroz, A. A. Leslie Gunatilaka, John R. Perfect, Leah E. Cowen

**Affiliations:** 1 Department of Molecular Genetics, University of Toronto, Toronto, Ontario, Canada; 2 Department of Medicine, Duke University Medical Center, Durham, North Carolina, United States of America; 3 Department of Molecular Genetics and Microbiology, Duke University Medical Center, Durham, North Carolina, United States of America; 4 SW Center for Natural Products Research & Commercialization, Office of Arid Lands Studies, The University of Arizona, Tucson, Arizona, United States of America; Carnegie Mellon University, United States of America

## Abstract

Fungal pathogens exploit diverse mechanisms to survive exposure to antifungal drugs. This poses concern given the limited number of clinically useful antifungals and the growing population of immunocompromised individuals vulnerable to life-threatening fungal infection. To identify molecules that abrogate resistance to the most widely deployed class of antifungals, the azoles, we conducted a screen of 1,280 pharmacologically active compounds. Three out of seven hits that abolished azole resistance of a resistant mutant of the model yeast *Saccharomyces cerevisiae* and a clinical isolate of the leading human fungal pathogen *Candida albicans* were inhibitors of protein kinase C (PKC), which regulates cell wall integrity during growth, morphogenesis, and response to cell wall stress. Pharmacological or genetic impairment of Pkc1 conferred hypersensitivity to multiple drugs that target synthesis of the key cell membrane sterol ergosterol, including azoles, allylamines, and morpholines. Pkc1 enabled survival of cell membrane stress at least in part via the mitogen activated protein kinase (MAPK) cascade in both species, though through distinct downstream effectors. Strikingly, inhibition of Pkc1 phenocopied inhibition of the molecular chaperone Hsp90 or its client protein calcineurin. PKC signaling was required for calcineurin activation in response to drug exposure in *S. cerevisiae*. In contrast, Pkc1 and calcineurin independently regulate drug resistance via a common target in *C. albicans*. We identified an additional level of regulatory control in the *C. albicans* circuitry linking PKC signaling, Hsp90, and calcineurin as genetic reduction of Hsp90 led to depletion of the terminal MAPK, Mkc1. Deletion of *C. albicans PKC1* rendered fungistatic ergosterol biosynthesis inhibitors fungicidal and attenuated virulence in a murine model of systemic candidiasis. This work establishes a new role for PKC signaling in drug resistance, novel circuitry through which Hsp90 regulates drug resistance, and that targeting stress response signaling provides a promising strategy for treating life-threatening fungal infections.

## Author Summary

Treating fungal infections is challenging due to the emergence of drug resistance and the limited number of clinically useful antifungal drugs. We screened a library of 1,280 pharmacologically active compounds to identify those that reverse resistance of the leading human fungal pathogen, *Candida albicans*, to the most widely used antifungals, the azoles. This revealed a new role for protein kinase C (PKC) signaling in resistance to drugs targeting the cell membrane, including azoles, allylamines, and morpholines. We dissected mechanisms through which PKC regulates resistance in *C. albicans* and the model yeast *Saccharomyces cerevisiae*. PKC enabled survival of cell membrane stress at least in part through the mitogen-activated protein kinase (MAPK) cascade in both species. In *S. cerevisiae*, inhibition of PKC signaling blocked activation of a key regulator of membrane stress responses, calcineurin. In *C. albicans*, Pkc1 and calcineurin independently regulate resistance via a common target. Deletion of *C. albicans PKC1* rendered fungistatic drugs fungicidal and reduced virulence in a mouse model. The molecular chaperone Hsp90, which stabilizes client proteins including calcineurin, also stabilized the terminal *C. albicans* MAPK, Mkc1. We establish new circuitry connecting PKC with Hsp90 and calcineurin and suggest a promising strategy for treating life-threatening fungal infections.

## Introduction

Microbial survival depends critically upon coordination of sensing environmental stimuli with control of the appropriate cellular responses. As a consequence, microbes have evolved elaborate mechanisms to sense and respond to diverse environmental stresses, including oxidative stress, osmotic stress, thermal stress, changes in pH, and nutrient limitation [1], [2]. Signal transduction cascades integrate recognition and response to these stresses as well as to challenges imposed by exposure to various small molecules that are a ubiquitous presence in the environment. Small molecules can have a dramatic effect on cellular signaling, mediate communication between microbes, or exert potentially lethal toxicity [3], [4], [5], [6], [7]. Many natural products are produced by microbes in competitive communities and can lead to selection for enhanced capacity to tolerate these agents. Since natural products and their derivatives are extensively used in medicine and agriculture [8], [9], the evolution of resistance to these agents can have profound consequences for human health.

The evolution of drug resistance in fungal pathogens poses considerable concern given that invasive fungal infections are a leading cause of human mortality worldwide, especially among immunocompromised individuals. The frequency of such infections is on the rise in concert with the growing population of patients with compromised immune systems due to chemotherapy, transplantation of organs or hematopoietic stem cells, or infection with HIV [10], [11]. The leading fungal pathogen of humans is *Candida albicans*, which ranks as the fourth most common cause of hospital acquired infectious disease and is associated with mortality rates approaching 50% [12], [13], [14]. There is a very limited repertoire of antifungal drugs with distinct targets for the treatment of fungal infections, in part due to the close evolutionary relationships between these eukaryotic pathogens and their hosts [15], [16]. Most of the antifungal drugs in clinical use target the biosynthesis or function of ergosterol, the main sterol of fungal membranes [2], [17], [18]. The therapeutic efficacy of most antifungal drugs is compromised by the emergence of drug resistant strains, superinfection with resistant strains, and by static rather than cidal activities that block fungal growth but do not eradicate the pathogen population. To improve clinical outcome it will be necessary to develop new antifungal drugs with different mechanisms of action and to discover drugs that improve the fungicidal activity of current antifungals.

The molecular basis of antifungal drug resistance is best characterized in the context of the azoles through studies with *C. albicans* and the model yeast *Saccharomyces cerevisiae*. The azoles have been the most widely deployed class of antifungal drugs for decades and inhibit lanosterol 14α-demethylase, encoded by *ERG11*, resulting in a block in ergosterol biosynthesis, the accumulation of a toxic sterol intermediate, and cell membrane stress [2], [17], [18]. The azoles are generally fungistatic against *Candida* species and many patients are on long-term therapy, creating favorable conditions for the emergence of resistance. Despite the evolutionary distance between *C. albicans* and *S. cerevisiae*, mechanisms of azole resistance are largely conserved [19]. Resistance can arise by mechanisms that minimize the impact of the drug on the fungus, such as the overexpression of multidrug transporters or alterations of the drug target that prevent the drug from inhibiting its target. Alternatively, resistance can arise by mechanisms that minimize drug toxicity, such as loss of function of the ergosterol biosynthetic enzyme Erg3, which blocks the production of the toxic sterol that would otherwise accumulate when the azoles inhibit Erg11. Recent studies have established that basal tolerance of wild-type strains and resistance due to mechanisms that mitigate drug toxicities without blocking the effect of the drug on the cell are often dependent upon stress responses that are critical for survival of azole-induced cell membrane stress [2], [18].

The key regulator of cellular stress responses implicated in both basal tolerance and resistance to azoles is Hsp90 [2], [18], [20]. Hsp90 is an essential molecular chaperone that regulates the stability and function of a diverse set of client proteins, many of which are regulators of cellular signaling [21], [22], [23]. In *S. cerevisiae* and *C. albicans*, inhibition of Hsp90 function blocks the rapid evolution of azole resistance and abrogates resistance that was acquired by diverse mutations [24], [25]. A central aspect of Hsp90's role in the emergence and maintenance of azole resistance is that it enables calcineurin-dependent stress responses that are required to survive the membrane stress exerted by azoles. In both yeast species, Hsp90 physically interacts with calcineurin keeping it in a stable conformation that is poised for activation [26], [27]. Inhibition of calcineurin function phenocopies inhibition of Hsp90 function, abrogating azole resistance of diverse mutants [24], [25]. This has led to the model that calcineurin is the key mediator of Hsp90-dependent azole resistance. Notably, in *C. albicans* both Hsp90 and calcineurin have recently been demonstrated to regulate resistance to the echinocandins, the only new class of antifungals to reach the clinic in decades; they inhibit the synthesis of (1,3)-β-D-glucan, a key component of the fungal cell wall [20], [27].

Another key cellular stress response pathway implicated in basal tolerance to antifungal drugs is the protein kinase C (PKC) cell wall integrity pathway, though it has only been implicated in tolerance to drugs targeting the cell wall. Central to the core of this signaling cascade is Pkc1, the sole PKC isoenzyme in *S. cerevisiae* that is essential under standard growth conditions and regulates maintenance of cell wall integrity during growth, morphogenesis, and response to cell wall stress [28], [29], [30], [31]. Signals are initiated by a family of cell surface sensors that are coupled to the small G-protein Rho1, which activates a set of effectors including Pkc1. Pkc1 signaling has been the focus of extensive study in *S. cerevisiae* where it is known to regulate multiple targets, most notably the mitogen-activated protein kinase (MAPK) cascade comprised of a linear series of protein kinases including the MAPKKK Bck1, the MAPKKs Mkk1/2, and the MAPK Slt2 that relays signals to the terminal transcription factors Rlm1 and Swi4/Swi6. While Pkc1 is not essential in *C. albicans* [32], the Pkc1-activated MAPK cascade is conserved in *C. albicans* with Bck1, Mkk2, and the Slt2 homolog Mkc1 [33]. In both species, components of the Pkc1 signaling cascade have been implicated in mediating tolerance to the stress exerted by the echinocandins that target the fungal cell wall [34], [35], [36], [37].

Here, we embarked on a drug screen of 1,280 pharmacologically active compounds to identify molecules that abrogate azole resistance of both an *S. cerevisiae* resistant mutant and a *C. albicans* clinical isolate. We identified a key role for PKC signaling in mediating crucial responses to azoles as well as to other drugs targeting the ergosterol biosynthesis pathway, including allylamines and morpholines. Pkc1 regulated responses to azoles at least in part via the MAPK cascade in both species via multiple downstream effectors. Strikingly, inhibition of Pkc1 function phenocopied inhibition of Hsp90 or calcineurin. In *S. cerevisiae*, compromise of PKC signaling blocked calcineurin activation in response to ergosterol biosynthesis inhibitors, providing a compelling mechanism for the impact on drug resistance. In *C. albicans*, we found that Pkc1 and calcineurin independently regulate resistance via a common target. The complexity of interactions linking PKC signaling, Hsp90, and calcineurin was further illuminated as genetic reduction of *C. albicans* Hsp90 resulted in destabilization of Mkc1 thereby blocking its activation. Deletion of *C. albicans PKC1* rendered the fungistatic ergosterol biosynthesis inhibitors fungicidal and attenuated virulence in a murine model of systemic disease. Our findings establish an entirely new role for PKC signaling in basal tolerance and resistance to ergosterol biosynthesis inhibitors, a novel mechanism through which Hsp90 regulates drug resistance, and that targeting Pkc1 provides a promising therapeutic strategy for life-threatening fungal infections.

## Results

### A screen of 1,280 pharmacologically active compounds identifies hits that abrogate azole resistance

**Figure 1:**
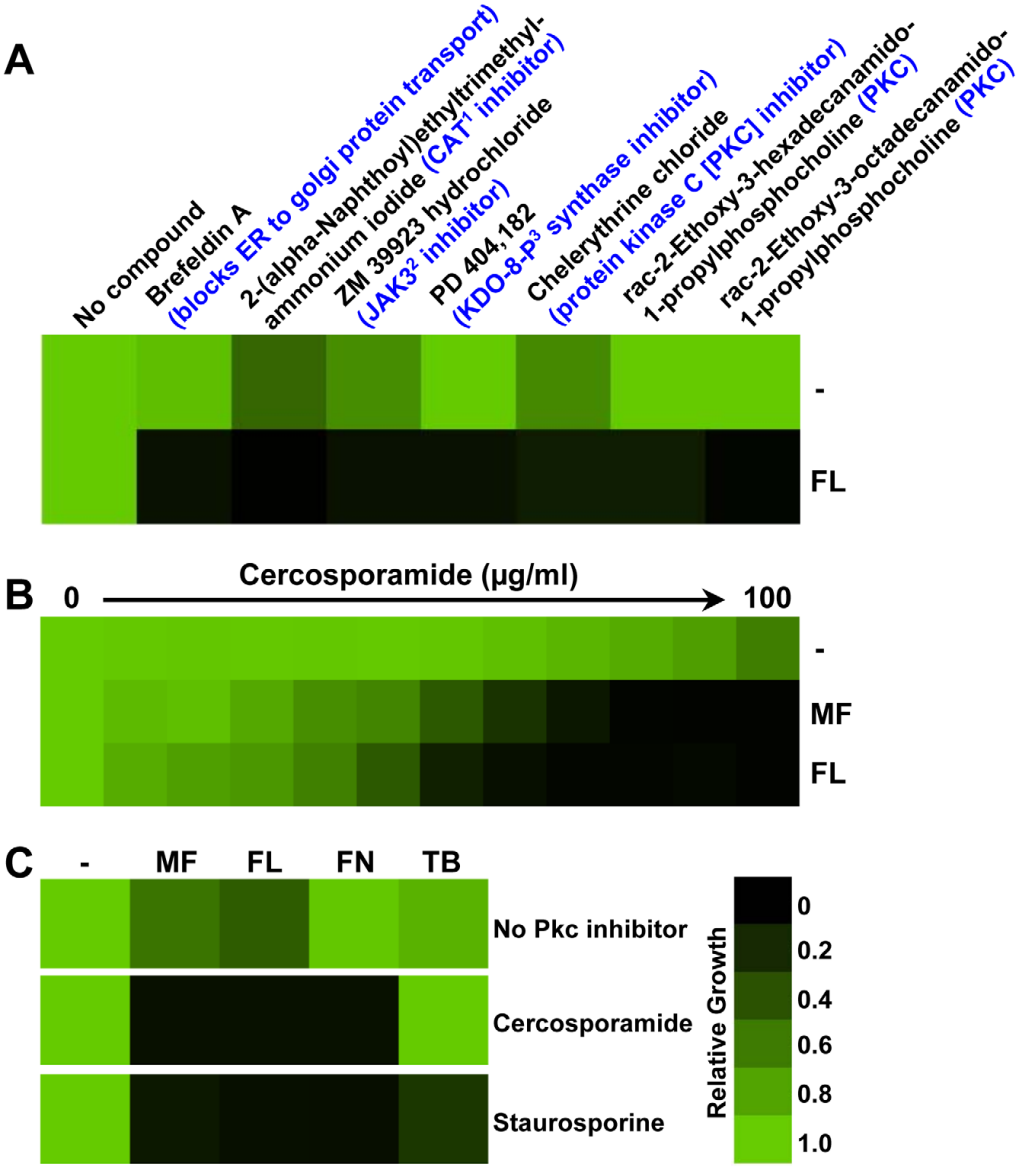
Pharmacological inhibition of PKC signaling enhances the efficacy of antifungal drugs targeting the cell membrane. (**A**) A drug screen identifies compounds that abrogate fluconazole (FL) resistance of a *Candida albicans* clinical isolate (CaCi-2). Seven compounds from the LOPAC^1280^ Navigator library had little toxicity on their own but enhanced the efficacy of FL against CaCi-2 when tested at 12.5 µM in RPMI medium with 2% glucose in the presence or absence of 8 µg/ml FL. Growth was measured by absorbance at 600 nm after 48 hours at 30°C. Optical densities were averaged for duplicate measurements and normalized relative to the no compound control (-) or FL-only control. Data was quantitatively displayed with colour using Treeview (see colour bar). The target or mode of action of each compound is indicated in blue. ^1^CAT = choline acetyltransferase; ^2^JAK3 = Janus kinase family protein; and ^3^KDO-8-P = 3-deoxy-D-manno-2-octulosonate-8-phosphate. (**B**) Pharmacological inhibition of Pkc1 with cercosporamide abrogates azole resistance and reduces echinocandin tolerance of CaCi-2 in an MIC assay. Assays were done in yeast peptone dextrose (YPD) with a fixed concentration of 2 µg/ml micafungin (MF) or 8 µg/ml FL, as indicated. Data was analyzed after 48 hours at 30°C as in part A. (**C**) Pkc1 inhibitors confer increased sensitivity to other ergosterol biosynthesis inhibitors. A fixed concentration of CaCi-2 cells was incubated in YPD with no antifungal (-), 2 µg/ml MF, 8 µg/ml FL, 2.5 µg/ml fenpropimorph (FN), or 2 µg/ml terbinafine (TB) and with the PKC inhibitors cercosporamide (100 µg/ml) or staurosporine (37.5 ng/ml), as indicated. Data was analyzed after 48 hours at 30°C as in part A.

To identify compounds that enhance the efficacy of the azole fluconazole we screened the LOPAC^1280^ Navigator library. Our initial screen used an *S. cerevisiae* strain with azole resistance due to deletion of *ERG3*. This resistance phenotype is exquisitely sensitive to perturbation of stress response pathways [24], [25]. To enhance the activity of library compounds, this azole-resistant mutant also harbored deletion of *PDR1* and *PDR3*, transcription factors that regulate the expression of numerous multidrug transporters which efflux structurally diverse compounds from the cell [38]. The library was initially screened at 25 µM in defined RPMI medium at 30°C in the presence of 8 µg/ml fluconazole, which reduces growth of this strain by less than 50% . The compounds that reduced growth by greater than or equal to 50% relative to the fluconazole-only controls were re-screened at 12.5 µM in the presence and absence of fluconazole to distinguish those that enhance the activity of fluconazole from those that are simply toxic on their own. This screen identified 185 compounds that enhanced the efficacy of fluconazole (data not shown). To prioritize compounds with synergistic activity with fluconazole against a clinical isolate of *C. albicans*, we then screened the 185 compounds at 12.5 µM for activity against an isolate from an HIV-infected patient undergoing fluconazole treatment, both in the presence and absence of fluconazole at 8 µg/ml. The capacity of this clinical isolate to grow in the presence of high concentrations of azole is critically dependent upon cellular stress responses [25], despite the fact that it has increased expression of the multidrug transporter Mdr1 relative to a drug-sensitive isolate recovered from the same patient at an earlier time point [39], [40], [41]. This secondary screen identified seven compounds that had little toxicity on their own but which enhanced the efficacy of fluconazole (Figure 1A). One hit from our screen, brefeldin A, was recently confirmed to exhibit potent synergy with antifungals against *Candida* and *Aspergillus* [42]. Strikingly, three of the seven hits were characterized as inhibitors of protein kinase C (PKC).

### Pharmacological inhibition of PKC enhances the efficacy of antifungal drugs targeting the cell membrane

PKC governs the cell wall integrity signaling pathway so named for its role in regulating cell wall integrity during growth, morphogenesis, and exposure to stress in fungi [29], [30], [31]. In both *S. cerevisiae* and *C. albicans*, the PKC signaling cascade is known to regulate cellular responses crucial for survival of exposure to antifungal drugs targeting the cell wall, such as the echinocandins [34], [35], [36], [37]. Since the PKC inhibitors identified in our screen were characterized in mammalian cells [43], [44], we next turned to other pharmacological inhibitors of PKC whose mode of action had been validated in fungi. Cercosporamide was identified as a selective Pkc1 inhibitor through *C. albicans* Pkc1-based high-throughput screening and was shown to exhibit potent synergy with echinocandins [45]. We purified cercosporamide from the fungus *Cercosporidium henningsii* following standard protocols [46]. As a positive control, we tested the impact of a concentration gradient of cercosporamide on growth in the presence of a fixed concentration of the echinocandin micafungin that causes less than 50% inhibition of growth on its own and confirmed that cercosporamide had the expected synergistic activity with micafungin against the clinical *C. albicans* isolate (Figure 1B). Using a comparable assay, we determined that cercosporamide also enhanced the activity of fluconazole (Figure 1B), validating the results from our screen. We further confirmed our pharmacological findings with another PKC inhibitor characterized in fungi, staurosporine [47], [48]. Both cercosporamide and staurosporine enhanced the efficacy of antifungals targeting the cell wall, micafungin, and those targeting the cell membrane (Figure 1C), including fluconazole and the morpholine fenpropimorph, which inhibits Erg2 and Erg24 [49]. While staurosporine enhanced the efficacy of another ergosterol biosynthesis inhibitor that inhibits Erg1 [49], the allylamine terbinafine, cercosporamide did not (Figure 1C). The lack of effect of cercosporamide on terbinafine tolerance is likely an artifact of an inactivating drug-drug interaction given that mutants that are hypersensitive to terbinafine are rendered resistant by cercosporamide (data not shown).

### Genetic validation that Pkc1 enables tolerance to drugs that affect the cell membrane via the MAPK cascade in *S. cerevisiae*

**Figure 2:**
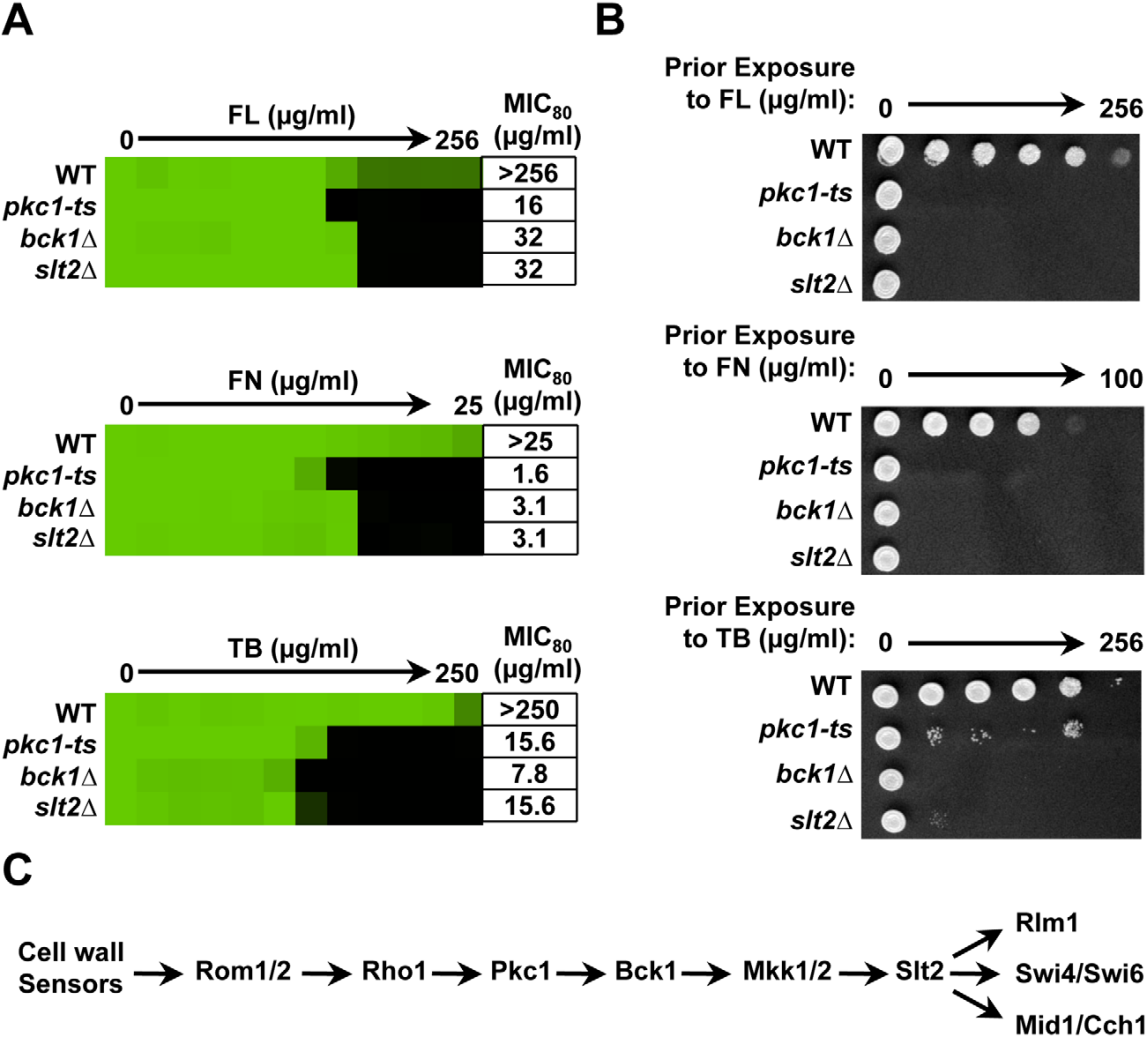
Pkc1 enables basal tolerance to ergosterol biosynthesis inhibitors via the MAPK cascade in *Saccharomyces cerevisiae*. (**A**) Drug tolerance of a wild-type (WT) strain (BY4741), a derivative (pkc1-ts) with a temperature sensitive *PKC1* allele, and derivatives with deletions of *BCK1* and *SLT2* are compared in MIC assays. Assays were performed in synthetic defined (SD) medium at 35°C. Data was analyzed after 48 hours as in Figure 1A. The minimum drug concentration that inhibits growth by 80% relative to the drug-free growth control (MIC_80_) is indicated for each strain. (**B**) Genetic compromise of Pkc1 creates a fungicidal combination with ergosterol biosynthesis inhibitors. MIC assays with two-fold dilutions of fluconazole (FL), fenpropimorph (FN), and terbinafine (TB) were performed in SD and incubated for 48 hours at 35°C. Cells from the MIC assays were spotted onto YPD medium and incubated at 30°C for 48 hours before plates were photographed. (**C**) Schematic of the *S. cerevisiae* Pkc1 cell wall integrity pathway.

In *S. cerevisiae*, *PKC1* is essential [50], thus we used a strain harboring only a temperature-sensitive (ts) *pkc1-3* allele [51] and assayed tolerance to three ergosterol biosynthesis inhibitors fluconazole, fenpropimorph, and terbinafine. Growth of the wild-type strain and the *pkc1-3* ts mutant was assayed over a gradient of drug concentrations relative to a drug-free control at either the permissive temperature (30°C) or at a more restrictive temperature, but where the *pkc1-3* ts mutant was still able to thrive in the absence of antifungals (35°C). At the permissive temperature, the wild type and the *pkc1-3* ts mutant had comparable tolerance to all three drugs tested (Figure S1A). At the restrictive temperature, the *pkc1-3* ts mutant was hypersensitive to all three drugs (Figure 2A). The same trend was observed when a dilution series of cells was spotted on solid medium with a fixed concentration of drug (Figure S1B). To determine if reduction of Pkc1 function rendered the fungistatic ergosterol biosynthesis inhibitors fungicidal we used tandem assays with an antifungal susceptibility test performed at the restrictive temperature followed by spotting onto rich medium without any inhibitors. The wild-type strain was able to grow on rich medium following exposure to all concentrations of drug tested (Figure 2B); compromise of Pkc1 function in the *pkc1-3* ts mutant enhanced cidality of all three drugs with the most severe effect for fluconazole and fenpropimorph. Thus, reduction of Pkc1 activity increases sensitivity to drugs targeting the cell membrane and enhances cidality of these otherwise fungistatic agents.

Despite the simple linear schematic commonly used to illustrate the architecture of the Pkc1 cell wall integrity pathway (Figure 2C), there is evidence for additional Pkc1 targets [30] and multiple cases of cross talk with other stress response pathways [28]. We next sought to determine if the effects of Pkc1 on tolerance to ergosterol biosynthesis inhibitors are due to signaling via the downstream MAPK cascade. *S. cerevisiae* mutants lacking the MAPKKK Bck1 or the terminal MAPK Slt2 were hypersensitive to all three ergosterol biosynthesis inhibitors tested in both a liquid antifungal susceptibility assay measuring growth of a fixed concentration of cells across a gradient of drug concentrations (Figure 2A) and a spotting assay of a dilution of cells on solid medium with a fixed concentration of drug (Figure S1B). Deletion of the MAPK components also rendered these fungistatic drugs fungicidal. Thus, Pkc1 enables tolerance to ergosterol biosynthesis inhibitors via the MAPK cascade in *S. cerevisiae*.

### Pkc1 enables tolerance to drugs that affect the cell membrane in part via the MAPK cascade in *C. albicans*

**Figure 3:**
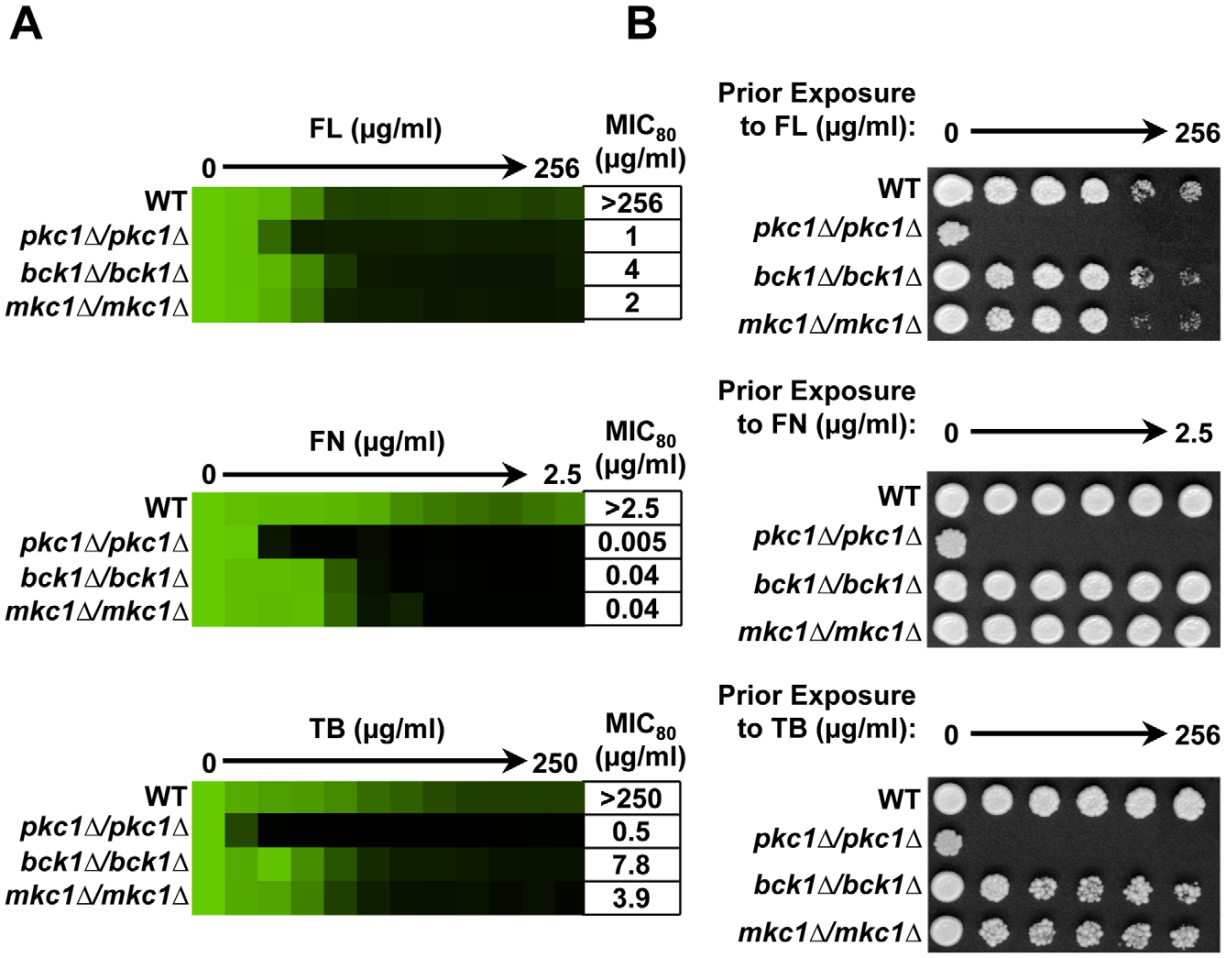
Pkc1 enables basal tolerance to ergosterol biosynthesis inhibitors in part via the MAPK cascade in *Candida albicans*. (**A**) Deletion of *PKC1*, *BCK1* or *MKC1* reduces tolerance to fluconazole (FL), fenpropimorph (FN), and terbinafine (TB) in MIC assays. Assays were performed in YPD medium at 35°C with strains derived from the WT SN95. Data was analyzed after 72 hours growth as in Figure 1A. The minimum drug concentration that inhibits growth by 80% relative to the drug-free growth control (MIC_80_) is indicated for each strain. (**B**) Deletion of *PKC1*, but not MAPK components, creates a fungicidal combination with the ergosterol biosynthesis inhibitors in *C. albicans*. MIC assays with four-fold dilutions of FL, FN, and TB were performed in YPD and incubated for 48 hours at 35°C. Cells from the MIC assays were spotted onto YPD medium and incubated at 30°C for 48 hours before plates were photographed.

In *C. albicans*, *PKC1* is not essential though it does share a high degree of sequence conservation with *S. cerevisiae PKC1* and has a conserved role in regulating cell wall integrity through a conserved MAPK cascade [32], [33]. To genetically validate the role of *C. albicans PKC1* in tolerance to drugs affecting the cell membrane, we constructed a *pkc1Δ/pkc1Δ* mutant. Homozygous deletion of *PKC1* rendered the strain hypersensitive to all three ergosterol biosynthesis inhibitors tested in liquid static susceptibility assays (Figure 3A) as well as on solid medium (Figure S2A). Comparable results were obtained in well-aerated shaking liquid cultures (data not shown). Restoring a wild-type *PKC1* allele under the control of the native promoter to the native locus restored drug tolerance (Figure S2). To determine if deletion of *C. albicans PKC1* renders the ergosterol biosynthesis inhibitors fungicidal, we used tandem assays with an antifungal susceptibility test followed by spotting onto rich medium without inhibitor. A strain with wild-type *PKC1* levels was able to grow on rich medium following exposure to all drug concentrations tested (Figure 3B). Homozygous deletion of *C. albicans PKC1* was cidal in combination with any dose of ergosterol biosynthesis inhibitor tested; no cells were able to grow on rich medium following exposure to the treatments. Thus, Pkc1 regulates crucial cellular responses for surviving the cell membrane stress exerted by antifungal drugs.

As an initial approach to assess whether the MAPK cascade was implicated in responses to drugs targeting the cell membrane, we monitored activation of the terminal MAPK in *C. albicans*. Mkc1 is known to be activated in response to distinct stress conditions including oxidative stress, changes in osmotic pressure, cell wall damage, and cell membrane perturbation [52]. To determine if Mkc1 is activated in response to ergosterol biosynthesis inhibitors we monitored Mkc1 phosphorylation using an antibody that detects dual phosphorylation on conserved threonine and tyrosine residues. Exposure to fluconazole, fenpropimorph, and terbinafine led to Mkc1 activation comparable to exposure to the cell wall damaging antifungal micafungin (Figure S3A). However, activation of signal transducers is not always coupled with functional consequences of their deletion. For example, Mkc1 is activated by exposure to hydrogen peroxide but is not required for survival of this stress [52].

To determine if the role of the MAPK cascade was conserved in *C. albicans*, we constructed homozygous deletion mutants lacking either the MAPKKK Bck1 or the terminal MAPK Mkc1 (homolog of *S. cerevisiae* Slt2). Homozygous deletion of either *BCK1* or *MKC1* rendered strains hypersensitive to fluconazole, fenpropimorph, and terbinafine (Figure 3A) but had negligible effect at elevated temperatures (Figure S3B). This stands in contrast to our results with *S. cerevisiae* that demonstrated an equivalent role of the MAPK cascade at all temperatures tested (Figure 2, Figure S1 and S4). While deletion of *C. albicans PKC1* rendered the ergosterol biosynthesis inhibitors fungicidal, deletion of *BCK1* or *MKC1* did not (Figure 3B). These results not only implicate the MAPK cascade in *C. albicans* but also suggest that alternate effectors downstream of Pkc1 are more important at elevated temperature and enable survival in the presence of ergosterol biosynthesis inhibitors.

### The role of targets downstream of the terminal MAPK in tolerance to ergosterol biosynthesis inhibitors

Effectors downstream of the terminal MAPK of the PKC signaling cascade have been well studied in *S. cerevisiae* and include both nuclear and cytoplasmic proteins. Slt2 is known to regulate activation of two transcription factors Rlm1 and SBF, which is comprised of Swi4 and Swi6 [30]. Rlm1 mediates the majority of the transcriptional output of cell wall integrity signaling, largely genes involved in cell wall biogenesis [53]. SBF drives cell cycle-specific transcription and is also regulated by Slt2 in response to cell wall stress (reviewed in [30]). Swi4 interacts directly with Slt2 and has additional roles in transcriptional regulation independent of the regulatory subunit Swi6 [54]. Slt2 translocates from the nucleus to the cytoplasm in response to cell wall stress [55]. Cytoplasmic Slt2 is required for activation of a high-affinity Ca^2+^ influx system in the plasma membrane that is comprised of two subunits, Cch1 and Mid1, in response to endoplasmic reticulum stress [56]. Activation of the Cch1-Mid1 channel leads to the accumulation of intracellular Ca^2+^ and activation of the protein phosphatase calcineurin [57].

**Figure 4:**
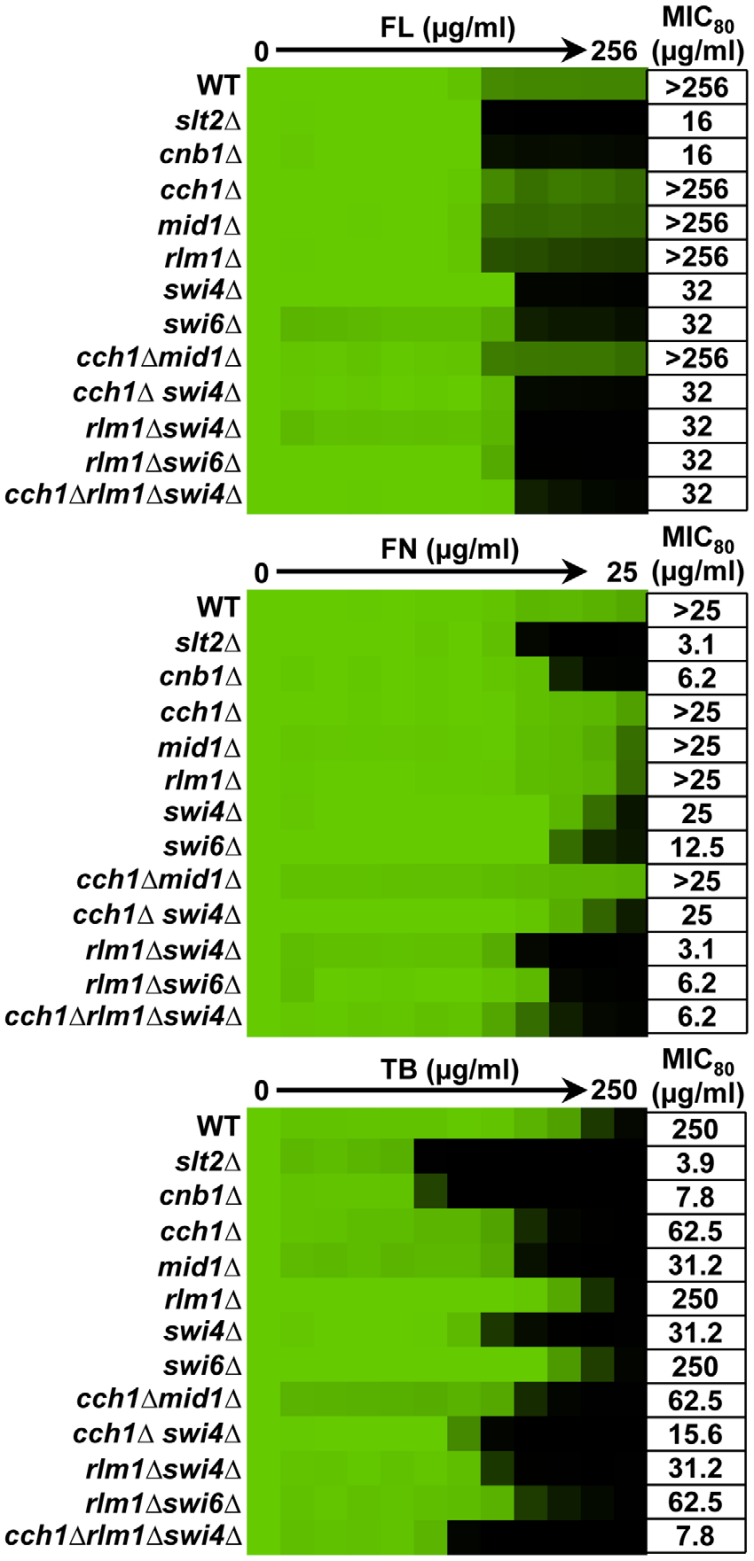
Distinct downstream effectors are important for tolerance of *S. cerevisiae* to different ergosterol biosynthesis inhibitors. To dissect the role of downstream effectors of Slt2 in tolerance to ergosterol biosynthesis inhibitors, we tested the impact of their deletion individually and in combination on drug susceptibility in an MIC assay. Data was analyzed after 72 hours at 35°C in SD medium as in Figure 1A. The minimum drug concentration that inhibits growth by 80% relative to the drug-free growth control (MIC_80_) is indicated for each strain.

To dissect the role of downstream effectors of Slt2 in ergosterol biosynthesis inhibitor tolerance, we tested the impact of their deletion individually and in combination on antifungal susceptibility. For reference, we included a strain lacking the regulatory subunit of calcineurin, *CNB1*, which is hypersensitive to ergosterol biosynthesis inhibitors [24]. For fluconazole, deletion of *RLM1*, *CCH1*, or *MID1* had negligible impact on tolerance while deletion of *SWI4* or *SWI6* rendered strains almost as sensitive as the *slt2Δ* mutant (Figure 4). To determine if there was redundancy among the downstream effectors, we constructed strains harboring deletion of multiple effectors. Deletion of *CCH1* phenocopies deletion of the entire channel and deletion of *SWI4* abolishes SBF function as well as Swi4-dependent transcription independent of SBF. Thus, combined deletion of *CCH1*, *SWI4*, and *RLM1* should eliminate the four known targets of Slt2 phosphorylation. No additional increase in sensitivity was observed in double or triple mutants. This suggests that the SBF transcription factor is of central importance for enabling responses to fluconazole. For fenpropimorph, deletion of *RLM1*, *CCH1*, or *MID1* had no impact on tolerance individually while deletion of *SWI4* or *SWI6* caused a partial increase in sensitivity (Figure 4). Deletion of *RLM1* in the context of the *swi4Δ* or *swi6Δ* mutants further increased fenpropimorph sensitivity. Deletion of *CCH1* in the mutant backgrounds had little additional impact. This suggests that SBF is the major determinant of fenpropimorph tolerance with *RLM1* enabling additional responses important in the absence of SBF. For tolerance to terbinafine, deletion of *RLM1* had no impact while deletion of *SWI4* caused a partial increase in sensitivity (Figure 4). Unlike tolerance to fluconazole and fenpropimorph, deletion of *SWI6* had negligible impact on terbinafine tolerance while deletion of *CCH1* or *MID1* caused a partial increase in sensitivity. Deletion of both *RLM1* and *CCH1* in the *swi4Δ* mutant caused an incremental increase in sensitivity (Figure 4). These results suggest that Swi4 enables terbinafine tolerance independent of the SBF complex and that Rlm1 and Cch1 mediate responses that are important in the absence of Swi4. Thus, distinct downstream effectors are important for tolerance of *S. cerevisiae* to different ergosterol biosynthesis inhibitors.

**Figure 5:**
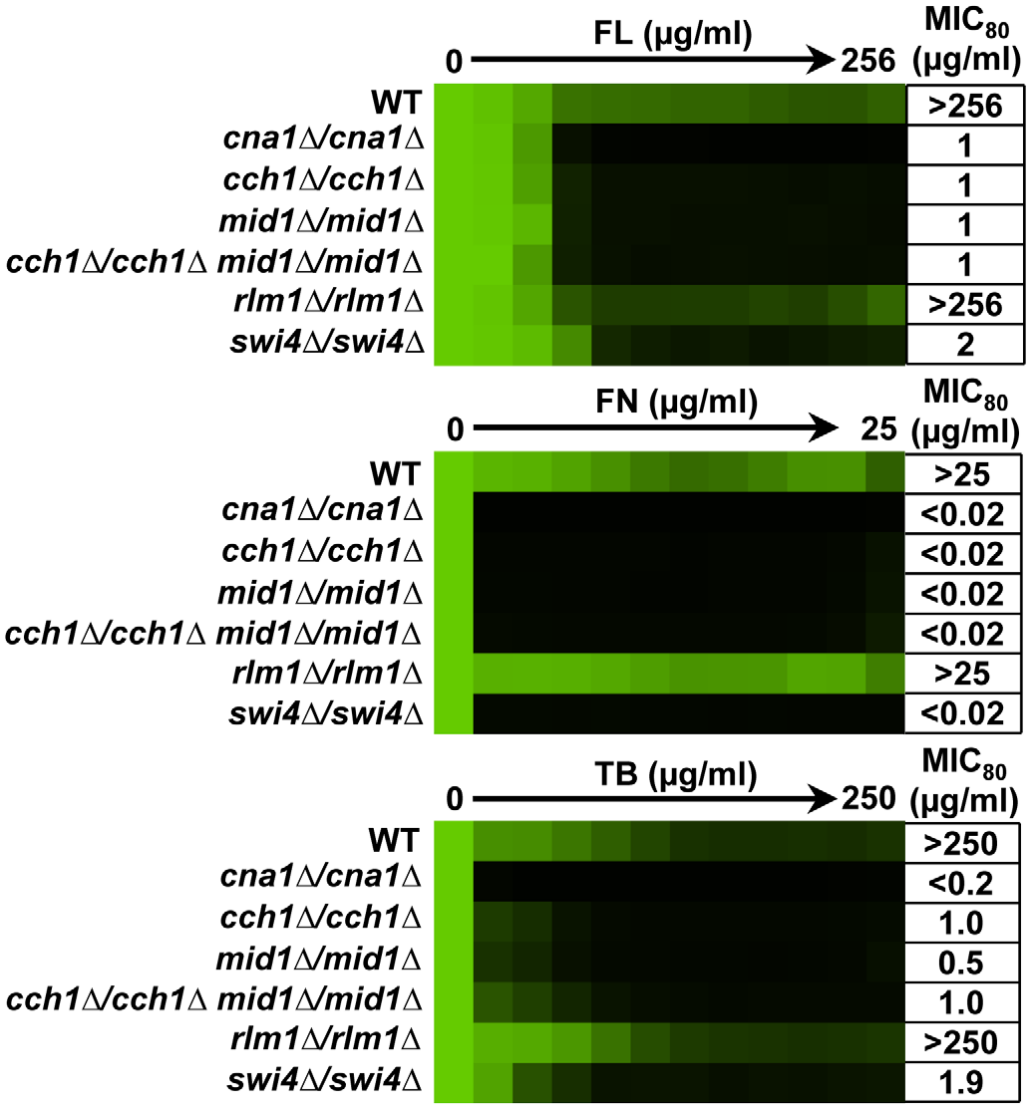
Swi4 and Cch1-Mid1 play critical roles in ergosterol biosynthesis inhibitor tolerance of *C. albicans*. Deletion of *SWI4* or components of the Cch1-Mid1 channel confer increased sensitivity to the ergosterol biosynthesis inhibitors in a MIC assay. Deletion of Rlm1 had no impact on drug sensitivity. A strain lacking the catalytic subunit of calcineurin (Cna1) is included for reference. Data was analyzed after 48 hours in YPD at 35°C as in Figure 1A. The minimum drug concentration that inhibits growth by 80% relative to the drug-free growth control (MIC_80_) is indicated for each strain.

Next, we tested a set of *C. albicans* mutants to determine if the role of the effectors downstream of the terminal MAPK of the PKC signaling cascade was conserved. As was the case with *S. cerevisiae*, deletion of *RLM1* on its own had no impact on tolerance to the ergosterol biosynthesis inhibitors (Figure 5), consistent with recent findings [58]. Deletion of *SWI4* rendered strains hypersensitive to all three ergosterol biosynthesis inhibitors tested (Figure 5). Deletion of *SWI6* or combined deletion of both *SWI4* and *SWI6* conferred a comparable increase in sensitivity (data not shown; unpublished strains generously provided by Catherine Bachewich), implicating the SBF complex in responses to drug-induced membrane stress. Deletion of *CCH1* or *MID1* individually or in combination had a comparable effect to deletion of *SWI4* rendering the strain hypersensitive to all three ergosterol biosynthesis inhibitors tested (Figure 5). Notably, *C. albicans cch1Δ/cch1Δ* and *mid1Δ/mid1Δ* mutants share some but not all phenotypes of a calcineurin mutant [59]. In terms of ergosterol biosynthesis inhibitor sensitivity, deletion of the gene encoding the catalytic subunit of calcineurin, *CNA1*, caused hypersensitivity akin to that of the *cch1Δ/cch1Δ* and *mid1Δ/mid1Δ* mutants for fluconazole and fenpropimorph but caused slightly greater sensitivity to terbinafine (Figure 5). Thus, in *C. albicans* both the SBF complex and the Cch1-Mid1 channel play critical roles in tolerance to drugs that target the cell membrane.

### PKC signaling enables calcineurin activation in response to ergosterol biosynthesis inhibitors via a mechanism distinct from Cch1-Mid1 in *S. cerevisiae*

**Figure 6:**
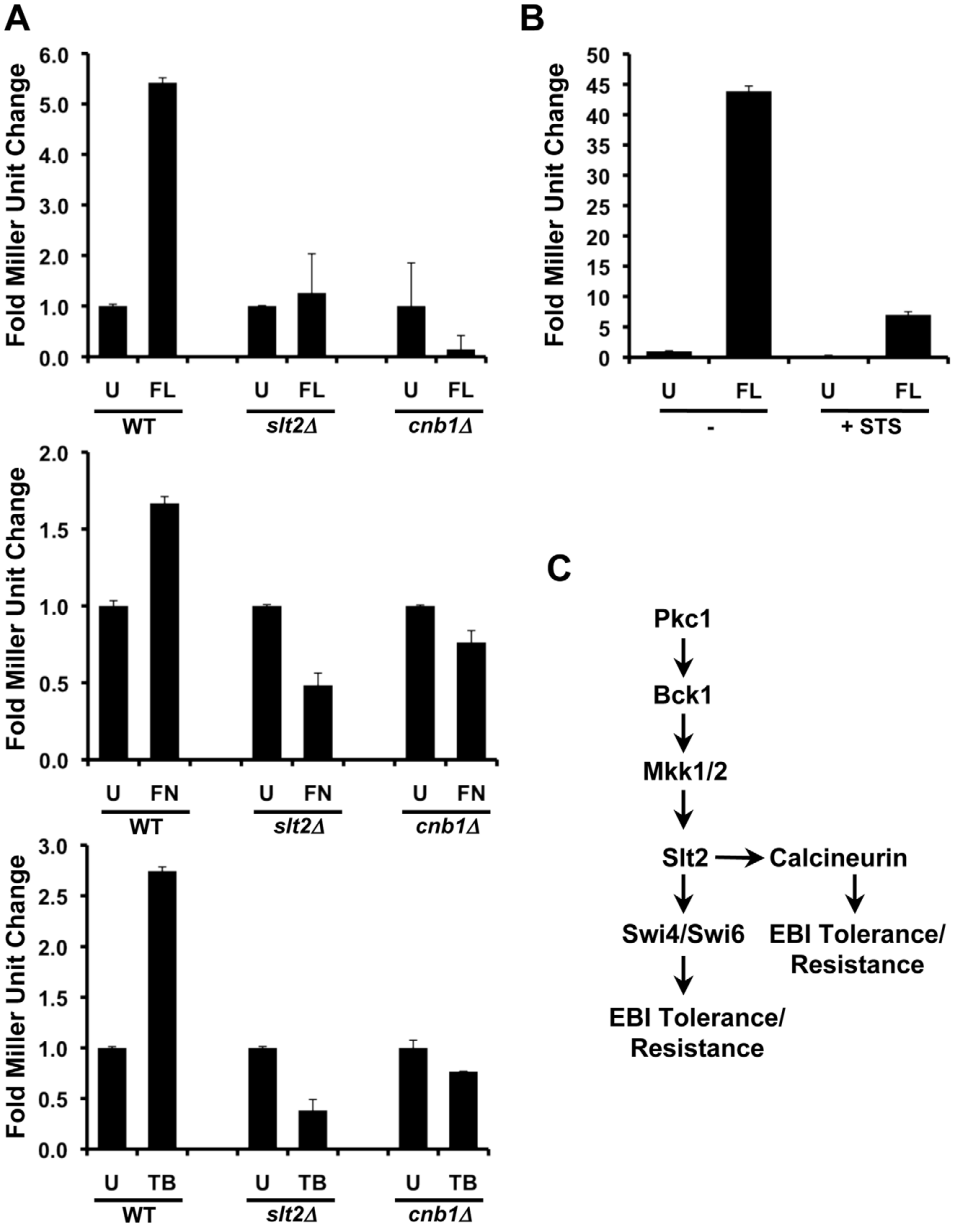
Compromising PKC-MAPK signaling blocks calcineurin activation in response to ergosterol biosynthesis inhibitors in *S. cerevisiae*. (**A**) Genetically compromising PKC-MAPK signaling by deleting *SLT2* blocks calcineurin activation monitored with a 4XCDRE-*lacZ* reporter. β-galactosidase activity was measured after incubation in SD medium for 24 hours without any antifungal (U) or in the presence of ergosterol biosynthesis inhibitors at the following concentrations: 16 µg/mL fluconazole (FL), 1 µg/mL fenpropimorph (FN), or 25 µg/mL terbinafine (TB). While the WT strain exhibited increased β-galactosidase activity in response to ergosterol biosynthesis inhibitors, deletion of *SLT2* or *CNB1* (which encodes the regulatory subunit of calcineurin) blocked calcineurin activation. Data are means ± SD for triplicate samples and are representative of two independent experiments. (**B**) Pharmacological inhibition of PKC signaling with staurosporine (STS) blocks calcineurin activation monitored with a 4XCDRE-*lacZ* reporter. β-galactosidase activity was measured after incubation in SD medium (-) or in SD with 2.5 µg/mL STS. Cells were then treated with 32 µg/mL FL or were left untreated (U). Data are means ± SD for triplicate samples and are representative of two independent experiments. (**C**) Simplified schematic of how *S. cerevisiae* Pkc1 regulates responses to ergosterol biosynthesis inhibitors (EBIs) important for basal tolerance and resistance.

Given calcineurin's established role in mediating drug-induced membrane stress responses [24], [25], [57] and that Slt2 has been shown to enable calcineurin activation by phosphorylating Cch1 [56], we tested whether calcineurin was activated in response to ergosterol biosynthesis inhibitors and whether deletion of Slt2 blocked this activation. To monitor calcineurin activation, we used a well-established reporter system that exploits the downstream effector Crz1, a transcription factor that is dephosphorylated upon calcineurin activation [60], [61]. Dephosphorylated Crz1 translocates to the nucleus, driving expression of genes with calcineurin-dependent response elements (CDREs) in their promoters. We used a reporter containing four tandem copies of CDRE and a *CYC1* minimal promoter driving *lacZ* [61]. We confirmed previous findings that fluconazole activates calcineurin ([27], [62] and Figure 6A). We also found that the other ergosterol biosynthesis inhibitors terbinafine and fenpropimorph activate calcineurin (*P*<0.001, ANOVA, Bonferroni's Multiple Comparison Test, Figure 6A). Deletion of *SLT2* completely blocked calcineurin activation in response to ergosterol biosynthesis inhibitors as did deletion of the regulatory subunit of calcineurin required for its activation, encoded by *CNB1* (*P*<0.001). Pharmacological inhibition of PKC signaling with staurosporine also blocked calcineurin activation (*P*<0.001, Figure 6B). The block in calcineurin activation was not an artifact of compromised viability as treatment conditions were optimized such that all cultures underwent comparable growth with equivalent protein yields. Given that the *slt2Δ* mutant is slightly more sensitive to ergosterol biosynthesis inhibitors than the mutant lacking calcineurin function, it is likely that Slt2 regulates responses to ergosterol biosynthesis inhibitors through additional targets. The *swi4Δ* mutant is less sensitive than the calcineurin mutant, suggesting that Slt2 regulates calcineurin function independently of Swi4 and that Swi4 regulates ergosterol biosynthesis inhibitor tolerance through additional targets (Figure 6C).

Given that deletion of *CCH1* and *MID1* had negligible effect on tolerance to fluconazole or fenpropimorph and only an intermediate effect on tolerance to terbinafine, it is likely that compromise of PKC signaling blocked calcineurin activation by a mechanism that is largely distinct from the Cch1-Mid1 channel. One possible mechanism is that the effects are transcriptional and mediated through a nuclear target of Slt2 such that inhibition of PKC signaling compromises the expression of calcineurin or *CRZ1*. However, deletion of *SLT2* did not reduce the expression of genes encoding any of the calcineurin subunits (*CNA1*, *CNA2* or *CNB1*) or *CRZ1* as measured by quantitative RT-PCR in the presence or absence of ergosterol biosynthesis inhibitor (*P*>0.05, ANOVA, Bonferroni's Multiple Comparison Test, Figure S5). Thus, PKC signaling enables calcineurin activation in response to ergosterol biosynthesis inhibitors by a mechanism that is largely distinct from the Cch1-Mid1 channel or transcriptional control of calcineurin.

### PKC signaling and calcineurin independently regulate tolerance to ergosterol biosynthesis inhibitors via a common target in *C. albicans*

**Figure 7:**
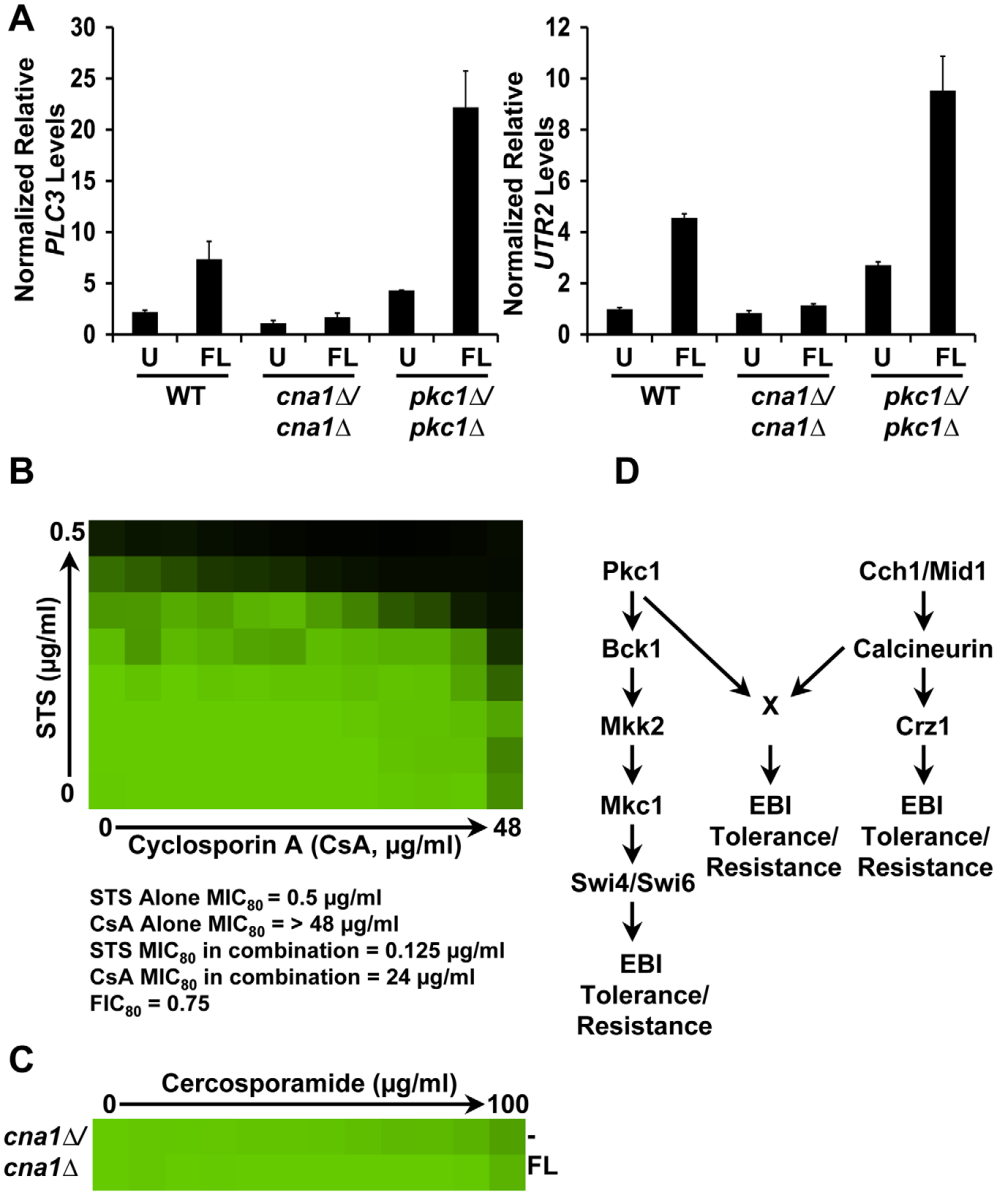
PKC signaling and calcineurin independently regulate tolerance to ergosterol biosynthesis inhibitors via a common target in *C. albicans*. (**A**) Deletion of *C. albicans PKC1* does not block EBI-induced activation of calcineurin. Transcript levels of two calcineurin-dependent genes, *PLC3* and *UTR2*, were measured by quantitative RT-PCR after growth in rich medium at 35°C for 6 hours without any antifungal (U) or with 16 µg/mL fluconazole (FL), as indicated. Transcripts were normalized to *GPD1*. Levels are expressed relative to the untreated wild-type samples, which were set to 1. Data are means ± SD for triplicate samples and are representative of two independent experiments. (**B**) Simultaneous inhibition of calcineurin and Pkc1 signaling does not synergistically decrease FL tolerance of a WT strain (SN95). A fractional inhibitory concentration (FIC) assay was carried out in YPD medium containing a fixed concentration of 0.5 µg/mL FL and gradients of the calcineurin inhibitor cyclosporin A (CsA) and the PKC inhibitor staurosporine (STS). Data was analyzed after growth at 35°C for 48 hours as in Figure 1A. The minimum concentration of STS or CsA that inhibits growth by 80% relative to the FL-only growth control (MIC_80_) individually or in combination is indicated along with the FIC. (**C**) FL tolerance of a mutant lacking the catalytic subunit of calcineurin is not sensitive to inhibition of PKC signaling. MIC assays were performed in YPD medium only (-) or YPD with a fixed concentration of 0.5 µg/mL FL. (**D**) Simplified schematic of how *C. albicans* Pkc1 regulates responses to ergosterol biosynthesis inhibitors (EBIs) important for basal tolerance and resistance.

In contrast to the minor impact of deletion of the *S. cerevisiae* Cch1-Mid1 channel, deletion of the *C. albicans* Cch1-Mid1 channel had nearly as great an effect as deletion of the catalytic subunit of calcineurin in response to fluconazole and fenpropimorph; for terbinafine the effect was partial (Figure 5). To test if the ergosterol biosynthesis inhibitors activate calcineurin and if inhibition of PKC signaling blocks this activation, we monitored transcript levels of two calcineurin-dependent genes, *PLC3* and *UTR2* [63]. In a wild-type strain, fluconazole activated calcineurin as measured by an increase in *PLC3* and *UTR2* transcript levels (*P*<0.05, ANOVA, Bonferroni's Multiple Comparison Test, Figure 7A). As expected, deletion of the catalytic subunit of calcineurin, *CNA1*, blocked the induction of *PLC3* and *UTR2* transcripts (*P*<0.01). Deletion of *PKC1* did not block induction of *PLC3* or *UTR2* indicating that impairment of PKC signaling does not block calcineurin activation (Figure 7A). At 35°C, conditions under which Pkc1 downstream effectors other than the MAPK cascade are more important in tolerance to ergosterol biosynthesis inhibitors, deletion of *PKC1* increased the magnitude of induction of *PLC3* and *UTR2* (*P*<0.001, Figure 7A). At 30°C, conditions under which the MAPK cascade mediates tolerance to ergosterol biosynthesis inhibitors, deletion of *PKC1* had no significant impact on calcineurin-dependent transcription (data not shown). Thus, drugs that inhibit ergosterol biosynthesis induce calcineurin activation in a manner that is independent of PKC signaling.

Next, we addressed alternative models that could explain the relationship between PKC signaling and calcineurin in *C. albicans* tolerance to ergosterol biosynthesis inhibitors. One possible model is that Pkc1 and calcineurin regulate tolerance through parallel but non-redundant pathways. This model leads to two predictions for ergosterol biosynthesis inhibitor tolerance: 1) there should be a synergistic effect of inhibiting both pathways simultaneously and 2) compromise of one pathway should confer increased sensitivity to inhibition of the other. To test the first prediction, we performed checkerboard assays in which a wild-type strain was exposed to a uniform concentration of fluconazole and a concentration gradient of both the calcineurin inhibitor cyclosporin A and the Pkc1 inhibitor staurosporine. There was no obvious synergy detected upon inhibition of both pathways in combination with fluconazole (Figure 7B). To assess this quantitatively we calculated the standard index of drug synergy, the fractional inhibitory concentration (FIC). The FIC value was 0.75 confirming that there was no synergy. To test the second prediction, we measured the impact of Pkc1 inhibition on fluconazole tolerance of a mutant lacking the catalytic subunit of calcineurin, *CNA1*. Growth of the *cna1Δ/cna1Δ* mutant was assessed in the absence or presence of the highest concentration of fluconazole that it could tolerate and with a gradient of the Pkc1 inhibitor cercosporamide. Fluconazole-sensitivity of the *cna1Δ/cna1Δ* mutant was not affected by cercosporamide (Figure 7C). The reciprocal was also true, such that the *pkc1Δ/pkc1Δ* mutant was not rendered hypersensitive to fluconazole by the calcineurin inhibitor cyclosporin A (data not shown). Thus, our results do not support either prediction of the model in which Pkc1 and calcineurin regulate ergosterol biosynthesis inhibitor tolerance through parallel pathways. We also ruled out the possibility that inhibition of calcineurin blocks PKC signaling as measured by levels of activated Mkc1 (data not shown). Taken together, these findings support a model in which Pkc1 and calcineurin independently regulate crucial responses to ergosterol biosynthesis inhibitors through a common target (Figure 7D). This target is not Crz1, the only well-characterized effector downstream of *C. albicans* calcineurin, given that transcription of the calcineurin-dependent genes *PLC3* and *UTR2* is mediated through the transcription factor Crz1 [63].

### Inhibition of PKC signaling phenocopies inhibition of Hsp90 reducing azole resistance of *C. albicans* clinical isolates and resistant mutants

**Figure 8:**
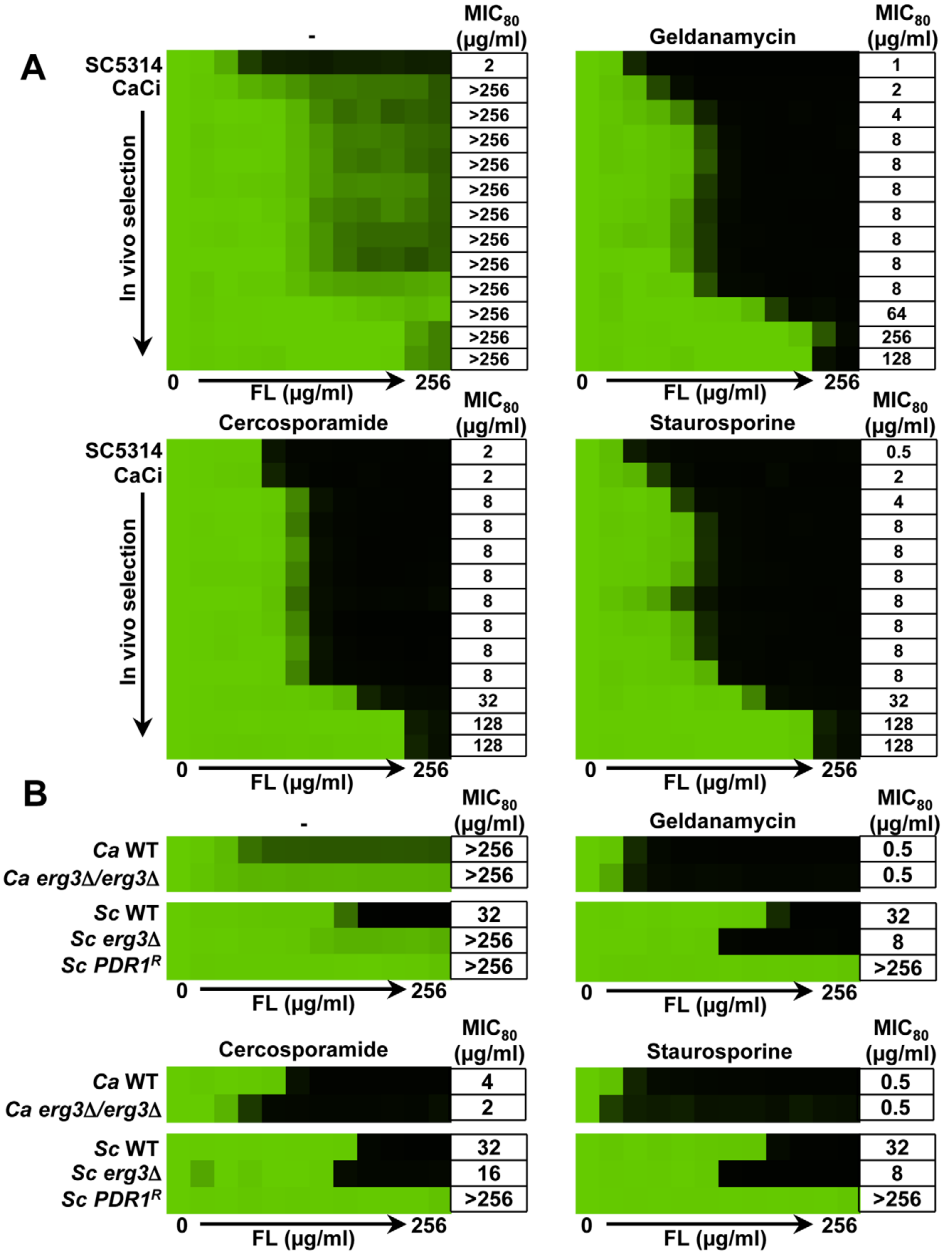
Inhibition of PKC signaling phenocopies inhibition of Hsp90 reducing azole resistance. (**A**) Fluconazole (FL) resistance of clinical isolates is abrogated by inhibition of Hsp90 or Pkc1. MIC assays were conducted in YPD medium with no inhibitor (-), with the Hsp90 inhibitor geldanamycin (5 µM), or with the Pkc1 inhibitors cercosporamide (12.5 µg/ml) or staurosporine (0.5 µg/ml). Clinical isolates (CaCi) are ordered sequentially with those recovered early in treatment at the top and those recovered late at the bottom; the FL-sensitive strain SC5314 is included as a control. Data was analyzed after growth for 48 hours at 30°C as in Figure 1A. (**B**) Inhibition of Hsp90 or Pkc1 abrogates FL resistance of both *S. cerevisiae* and *C. albicans erg3* mutants. Inhibition of Hsp90 or Pkc1 has no effect on the FL resistance of a *S. cerevisiae* strain (*PDR1^R^*) that overexpresses multiple drug efflux pumps due to an activating mutation in the transcription factor Pdr1. FL MIC assays were carried out in YPD medium only (-) or in YPD with fixed concentrations of: geldanamycin (*Sc*: 5 µM; *Ca*: 0.625 µM), cercosporamide (*Sc*: 50 µg/ml; *Ca*: 25 µg/ml), or STS (*Sc*: 0.625 µg/ml; *Ca*: 0.3125 µg/ml). Data was analyzed after growth for 48 hours at 30°C as in Figure 1A. The minimum drug concentration that inhibits growth by 80% relative to the no-FL growth control (MIC_80_) is indicated for each strain.

To determine if the role of PKC signaling in tolerance to drugs targeting the cell membrane was conserved in the context of *bona fide* drug resistance, we turned to *C. albicans* clinical isolates and resistant mutants (Figure 8). We tested the impact of two structurally unrelated Pkc1 inhibitors, cercosporamide and staurosporine, on azole susceptibility of a series of *C. albicans* isolates that evolved fluconazole resistance in a human host [40]. The isolates shown begin with the second isolate in the series, which is the first with elevated resistance and increased expression of the multidrug transporter Mdr1 [39], [40], [41]. Azole resistance of this series is known to have evolved from a state of dependence on calcineurin and Hsp90 to a state of independence and this change is associated with the accumulation of additional mutations [25]. The third isolate from the bottom (Figure 8A) has mutation (R467K) and increased expression of the azole target Erg11; the last two isolates, which show the least of effect of Hsp90 inhibition on resistance, also have increased expression of the multidrug transporter Cdr1 [39], [40], [41]. Inhibition of Pkc1 had a strikingly similar impact on azole resistance to inhibition of Hsp90 or calcineurin, reducing resistance of isolates recovered early during treatment to a greater extent than those recovered late during treatment (Figure 8A).

To further explore the relationship between stress response signaling and classic resistance mechanisms such as mutation of the drug target and overexpression of multidrug transporters, we characterized additional clinical isolates and laboratory-derived mutants. We tested an additional five sets of clinical isolates for which we had one isolate recovered early during azole treatment and one recovered later. In all cases, inhibition of Pkc1 phenocopied inhibition of Hsp90, with the least effect on azole resistance of isolates that overexpressed the multidrug transporter Cdr1 (Figure S6). Since the clinical isolates often harbor multiple mechanisms of resistance, we also tested specific laboratory-derived resistant mutants. Inhibition of Pkc1 abolished resistance of laboratory-derived *C. albicans* and *S. cerevisiae erg3* loss-of-function mutants (Figure 8B), as does inhibition of Hsp90 or calcineurin [24], [25]. In contrast, inhibition of Pkc1 did not affect *S. cerevisiae* resistance due to an activating mutation in the transcription factor Pdr1 that causes overexpression of multidrug transporters including Pdr5 (Figure 8B), as was the case with inhibition of Hsp90 [25]. We previously confirmed that genetic compromise of Hsp90 function does not affect resistance due to overexpression of Pdr5, confirming that the stability of this resistance phenotype is not due to Hsp90 inhibitors being pumped out of the cell [25]. Given the equivalent impact on azole resistance of Pkc1 inhibitors and Hsp90 inhibitors with diverse mutants, this strongly suggests that the stability of resistance of the Pdr1 mutant cannot be attributed to Pkc1 inhibitors being pumped out of the cell. Thus, inhibition of PKC signaling phenocopies inhibition of Hsp90 or its client protein calcineurin, reducing resistance of clinical isolates and specific resistant mutants. These results are consistent with the circuitry connecting PKC signaling and calcineurin delineated above and may also suggest an additional functional connection between Hsp90 and PKC signaling in regulating responses to ergosterol biosynthesis inhibitors.

### Genetic depletion of Hsp90 destabilizes *C. albicans* Mkc1

**Figure 9:**
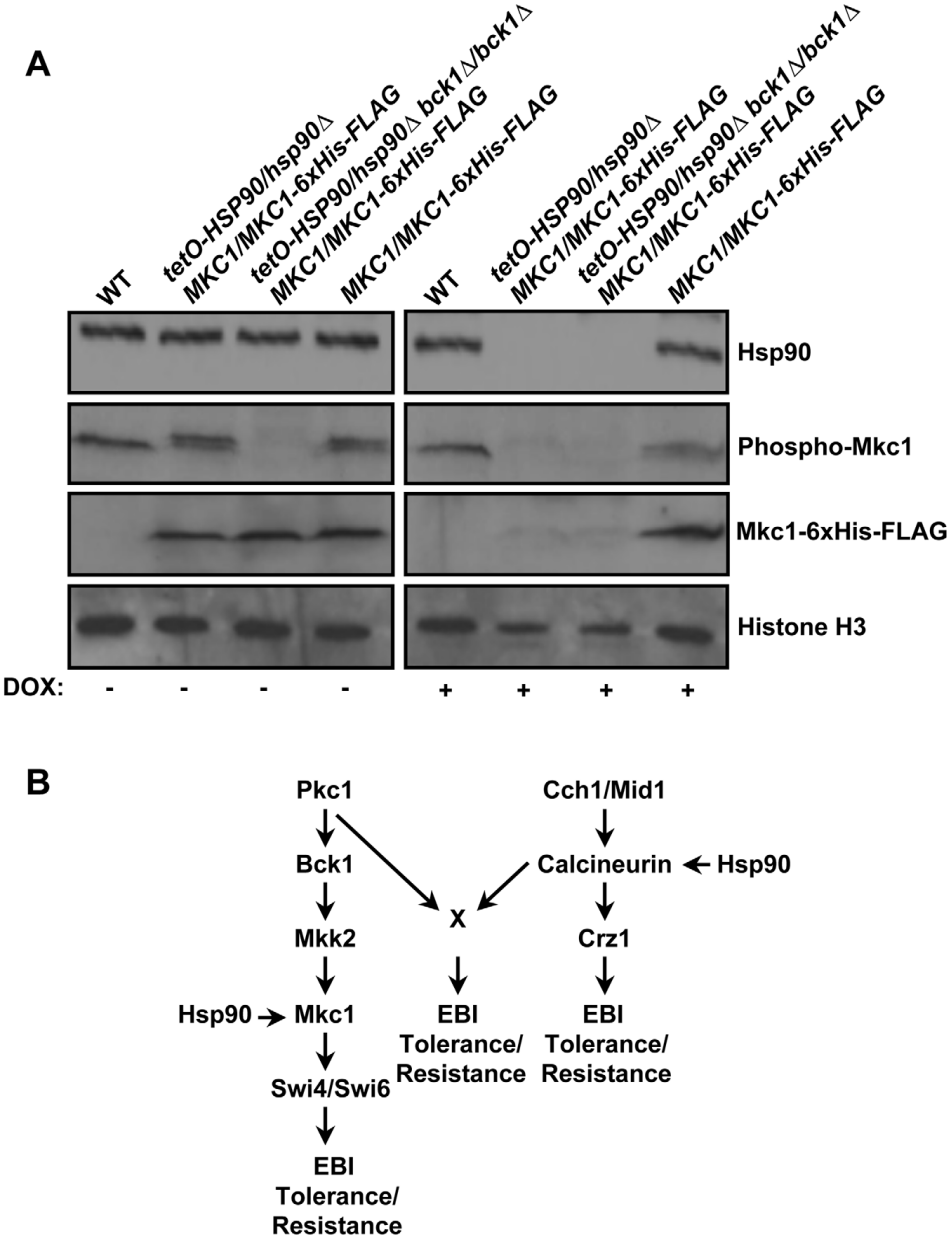
Hsp90 stabilizes the terminal MAPK Mkc1 in *C. albicans*. (**A**) Genetic reduction of Hsp90 levels results in depletion of Mkc1. In strains where the sole allele of *HSP90* is under the control of a tetracycline repressible promoter (*tetO*), transcription of *HSP90* can be repressed by tetracycline or the analog doxycycline (DOX). One allele of *MKC1* was C-terminally 6xHis-FLAG tagged for monitoring total levels of Mkc1. The MAPKKK Bck1 was deleted to block phosphorylation of Mkc1. Cells were grown with or without DOX (20 µg/ml) before being treated for 3 hours with 50 µg/ml terbinafine (TB) to elicit phosphorylation of Mkc1. Total protein was resolved by SDS-PAGE and blots were hybridized with α-Hsp90, α-His_6_ to monitor total Mkc1 levels, α-phospho p44/42 MAPK to monitor dually phosphorylated Mkc1 levels, and α-H3 as a loading control. (**B**) Simplified schematic of how *C. albicans* Hsp90 governs responses to ergosterol biosynthesis inhibitors (EBIs) important for basal tolerance and resistance by regulating both Pkc1-MAPK signaling and calcineurin signaling.

While our findings already establish a link between PKC signaling and calcineurin-mediated stress responses, we next explored the possibility of yet another functional connection between Hsp90 and PKC signaling. In *S. cerevisiae*, Hsp90 binds exclusively to the activated form of Slt2 and enables Slt2-mediated activation of the downstream target Rlm1 [64]. To determine if the connection between Hsp90 and the terminal MAPK is conserved in *C. albicans*, we tested the impact of genetic depletion of *C. albicans HSP90* on Mkc1 levels and activation status. To deplete Hsp90, we used a strain with its only *HSP90* allele under the control of a doxycycline repressible promoter [65]. To monitor total Mkc1 levels, this kinase was tagged at the C-terminus using a 6x-histidine and FLAG epitope tag. The Mkc1-6xHis-FLAG protein was functional and sufficient to confer wild-type tolerance to ergosterol biosynthesis inhibitors (Figure S7). To determine whether Hsp90 stabilized only the activated form of Mkc1, we used a strain lacking the upstream MAPKKK required for Mkc1 activation, Bck1. All strains were grown in the presence of terbinafine to induce Mkc1 activation. In the absence of doxycycline (Figure 9A, left panel), all strains had comparable levels of Hsp90 as measured relative to a histone H3 loading control. All strains also had comparable levels of activated dually-phosphorylated Mkc1, with the exception of the strain lacking Bck1 in which Mkc1 activation was blocked. Total Mkc1 levels monitored by a 6X-histidine antibody were comparable for the three strains harboring the tagged protein. In the presence of doxycycline (Figure 9A, right panel), Hsp90 levels were depleted only in the strains with the repressible promoter. Depletion of Hsp90 resulted in a corresponding depletion of total Mkc1 levels, even in the strain lacking Bck1 in which Mkc1 remains in the inactivate state. Depletion of Hsp90 did not affect *MKC1* transcript levels as measured by quantitative RT-PCR (*P*>0.05, ANOVA, Bonferroni's Multiple Comparison Test, Figure S8), confirming that the chaperone influences Mkc1 stability at the protein level. Thus, Hsp90 stabilizes Mkc1 independent of its activation status and thereby regulates PKC signaling, providing a new mechanism through which Hsp90 regulates drug-induced membrane stress responses (Figure 9B).

### Deletion of *C. albicans PKC1* attenuates virulence in a murine model of systemic disease

**Figure 10:**
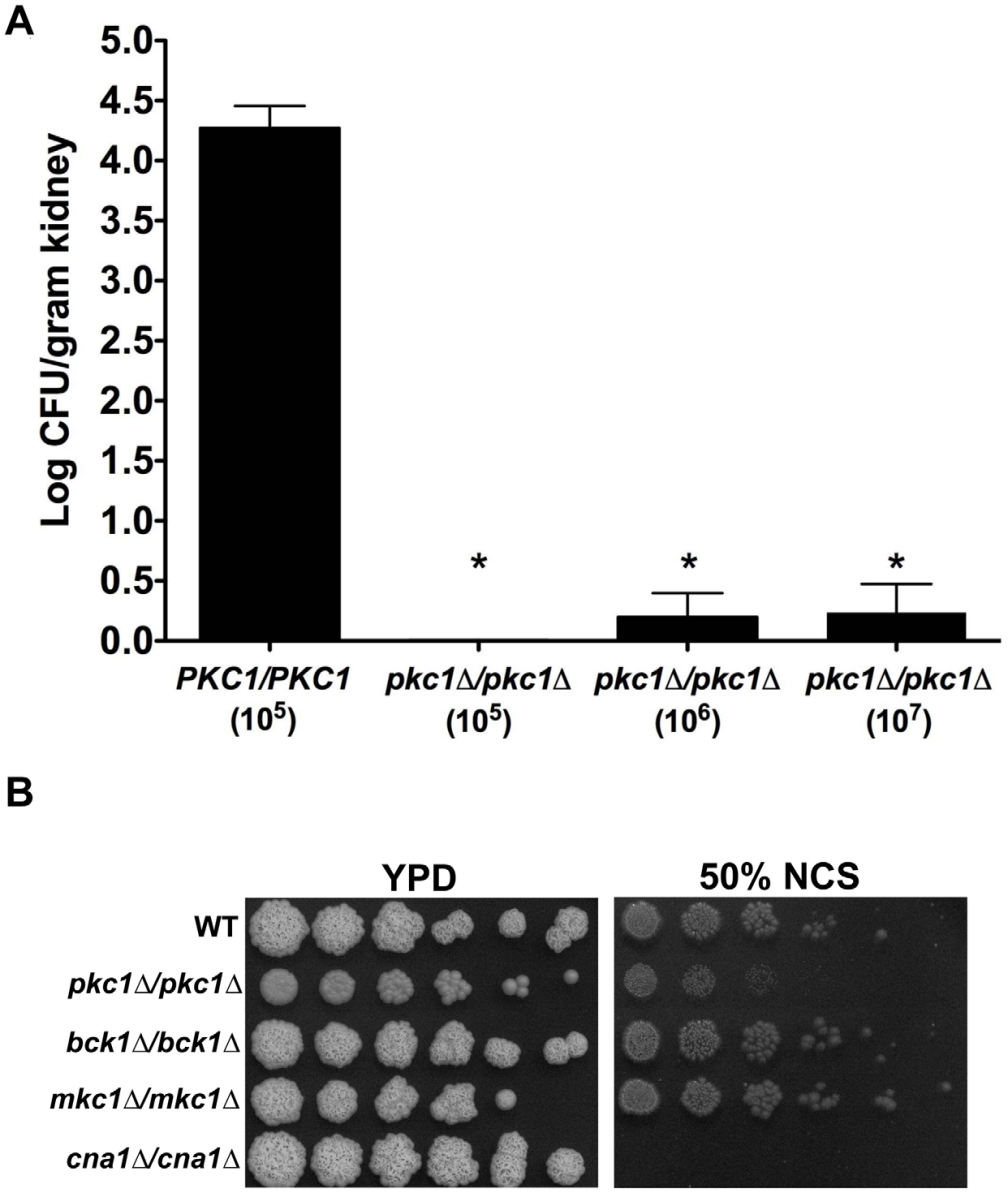
Deletion of *C. albicans PKC1* attenuates virulence in a murine model by targets distinct from calcineurin. (**A**) CD1 mice were infected with an inoculum of the wild-type strain (SN95) of 1×10^5^ CFU or inoculum of the *pkc1Δ/pkc1Δ* mutant of 1×10^5^ CFU, 1×10^6^ CFU, or 1×10^7^ CFU. Despite the higher innoculum used, deletion of *PKC1* resulted in a dramatic reduction of kidney fungal burden. Asterisks indicate *P*<0.001 (ANOVA, Bonferroni's Multiple Comparison Test). (**B**) Deletion of *PKC1*, but not components of the MAPK cascade, results in a modest increase in sensitivity to serum compared to the hypersensitivity of a mutant lacking the catalytic subunit of calcineurin, Cna1. Cells were spotted in fivefold dilutions (from 1×10^7^ cells/ml for *pkc1Δ/pkc1Δ*; from 1×10^6^ cells/ml for other strains) onto solid YPD medium with 50% new calf serum (NCS), as indicated. Plates were photographed after 72 hours growth at 35°C.

Given that deletion of *PKC1* enhances the efficacy of antifungal drugs, we next explored the therapeutic efficacy in a well-established murine model in which fungal inoculum is delivered by tail vein injection and progresses from the bloodstream to deep-seated infection of major organs, most notably the kidney [27], [65], [66]. We compared kidney fungal burden of mice infected with either a wild-type strain or a *pkc1Δ/pkc1Δ* mutant. The average kidney fungal burden in mice infected with 1×10^5^ CFUs of the wild-type parental strain was 4.34+/−0.54 log CFU per gram of kidney (Figure 10A). In stark contrast, the kidneys of mice infected with 1×10^5^ CFUs of the *pkc1Δ/pkc1Δ* mutant were sterile (Figure 10A). To determine if infection with higher inocula of the *pkc1Δ/pkc1Δ* mutant would lead to sufficient kidney fungal burden to enable assessment of antifungal efficacy *in vivo*, we tested the impact of infection with 10-fold and 100-fold higher inocula. Mice infected with 1×10^6^ or 1×10^7^ CFUs of the *pkc1Δ/pkc1Δ* mutant demonstrated significantly reduced fungal burden relative to those infected with only 1×10^5^ CFUs of the wild-type strain (*P*<0.001, ANOVA, Bonferroni's Multiple Comparison Test). The average kidney fungal burden in mice infected with 1×10^6^ or 1×10^7^ cells of the *pkc1Δ*/*pkc1Δ* mutant was 0.19+/−0.66 and 0.23+/−0.67 log CFU per gram of kidney, respectively. Thus, while *C. albicans PKC1* is dispensable for growth under standard conditions *in vitro* it is required for proliferation and infection in a murine model, providing evidence for a key role of this stress response regulator in virulence. While the attenuated virulence of the *pkc1Δ/pkc1Δ* mutant precluded straightforward studies to determine if compromising Pkc1 enhances the efficacy of antifungal drugs *in vivo*, it provides compelling support for therapeutic potential of compromising fungal Pkc1.

Given our findings that Pkc1 and calcineurin affect drug resistance via a common target in *C. albicans* (Figure 7), it is possible that Pkc1-mediated signaling may influence virulence by a target in common with calcineurin, which is known to be required for *C. albicans* virulence [67], [68], [69]. Calcineurin mutants are hypersensitive to calcium present in serum and are unable to survive transit through the bloodstream [68]. However, while a mutant lacking the catalytic subunit of calcineurin was unable to survive on medium containing 50% serum, the *pkc1Δ/pkc1Δ* mutant exhibited only an intermediate reduction in viability and the *mkc1Δ*/*mkc1Δ* and *bck1Δ*/*bck1Δ* mutants grew as well as the wild type (Figure 10B). Further, the *pkc1Δ/pkc1Δ* mutant grew as well as the wild-type strain in liquid serum while the calcineurin mutant was inviable (data not shown). These results suggest that Pkc1 exerts powerful control over *C. albicans* virulence by means of targets distinct from calcineurin.

## Discussion

Our results establish a new role for the PKC signal transduction cascade in resistance to drugs targeting the cell membrane in the model yeast *S. cerevisiae* and the fungal pathogen *C. albicans*. Three out of seven hits from our screen of 1,280 pharmacologically active compounds for those that abrogate azole resistance are classified as inhibitors of PKC, suggesting a central role for this cellular regulator in azole resistance (Figure 1). Pharmacological inhibition of Pkc1 with two additional structurally distinct PKC inhibitors whose mode of action has been validated in fungi or genetic compromise of Pkc1 function enhances sensitivity to azoles as well as other drugs targeting ergosterol biosynthesis, including allylamines and morpholines (Figures 1, 2 and 3). Pkc1 regulates responses to ergosterol biosynthesis inhibitors at least in part through the MAPK cascade in both species (Figures 2 and 3). In *S. cerevisiae*, signaling through the MAPK cascade is required for calcineurin activation suggesting that PKC signaling regulates crucial responses to ergosterol biosynthesis inhibitors through calcineurin in this species (Figure 6). In *C. albicans*, Pkc1 and calcineurin independently regulate responses to ergosterol biosynthesis inhibitors via a common target (Figure 7). Inhibition of Pkc1 phenocopies inhibition of calcineurin or Hsp90, reducing drug resistance of clinical isolates of *C. albicans* (Figure 8 and S6). We establish an additional level of regulatory complexity in the cellular circuitry linking PKC signaling, Hsp90, and calcineurin in that genetic reduction of *C. albicans* Hsp90 results in destabilization of the terminal MAPK, Mkc1, thereby blocking PKC signaling (Figure 9). This suggests that Hsp90 regulates basal tolerance and resistance to ergosterol biosynthesis inhibitors through Mkc1 in addition to the established connection with calcineurin. Our findings that compromising Pkc1 renders fungistatic drugs fungicidal (Figures 2 and 3) and attenuates virulence of *C. albicans* (Figure 10) suggest broad therapeutic potential.

The role of PKC signaling in basal tolerance and resistance to drugs targeting the cell membrane expands the repertoire of stress responses that depend upon this signal transduction cascade. In *S. cerevisiae*, it was previously appreciated that PKC signaling is required for basal tolerance to echinocandins, which target cell wall synthesis [35], [36]. This tolerance requires activation of the terminal MAPK Slt2 to drive Rlm1-dependent transcription of cell wall genes [36]. In *C. albicans*, the PKC pathway is activated by diverse stresses [52] and works in concert with calcineurin and the high osmolarity glycerol pathway to regulate chitin synthesis, which can enhance tolerance to echinocandins [37], [70]. As is the case with drugs compromising cell wall integrity, drugs targeting the cell membrane activate the terminal MAPK in the PKC cascade (Figure S3A). The role of PKC signaling in tolerance to drugs targeting the cell wall and the cell membrane raises the possibility that induction of cell membrane stress by ergosterol biosynthesis inhibitors could induce cell wall stress indirectly. This is consistent with the thought that the sensors involved in PKC cell wall integrity signaling are receptors that respond to changes in the structure of the cell membrane [71]. Despite the commonalities, the downstream regulation mediating responses to these different stresses diverge. Response to cell wall stress is largely dependent on the transcription factor Rlm1 [36], while regulation of cell membrane stress responses is largely independent of Rlm1. In *S. cerevisiae*, distinct downstream effectors contribute to tolerance to different ergosterol biosynthesis inhibitors (Figure 4). For fluconazole, the SBF transcription factor (Swi4/Swi6) is of central importance. For fenpropimorph, the SBF complex again is a major determinant with Rlm1 enabling responses important in the absence of SBF. For terbinafine, Swi4 enables tolerance largely independent of SBF and Rlm1 and Cch1-Mid1 mediate responses important in the absence of Swi4. These differences may be due to ergosterol depletion combined with the specific sterol that accumulates when ergosterol biosynthesis is inhibited at different points. In *C. albicans*, SBF and Cch1-Mid1 confer tolerance to all three ergosterol biosynthesis inhibitors tested suggesting that the point of inhibition of ergosterol biosynthesis has less impact than for *S. cerevisiae*.

The circuitry downstream of Pkc1 mediating membrane stress responses has been rewired considerably between *S. cerevisiae* and *C. albicans*. For *S. cerevisiae*, deletion of components of the MAPK cascade confers hypersensitivity to ergosterol biosynthesis inhibitors at all temperatures tested (Figure 2 and Figures S1 and S4). For *C. albicans*, deletion of components of the MAPK cascade confers hypersensitivity to ergosterol biosynthesis inhibitors at 30°C (Figure 3) but not at 35°C (Figure S3B), suggesting that the MAPK cascade is a key mediator of Pkc1-dependent cell membrane stress responses but that alternate downstream effectors play a dominant role in *C. albicans* at elevated temperature. The importance of alternate downstream effectors of Pkc1 in *C. albicans* is further emphasized as deletion of *PKC1* renders fungistatic ergosterol biosynthesis inhibitors fungicidal, while deletion of MAPK components does not (Figure 3B). Our findings highlight another divergence between the two species. While inhibition of PKC signaling blocks calcineurin activation in response to ergosterol biosynthesis inhibitors in *S. cerevisiae* (Figure 6), this is not the case in *C. albicans*. Rather, our results suggest that Pkc1 and calcineurin independently regulate responses to ergosterol biosynthesis inhibitors via a common target in *C. albicans* (Figure 7). As with PKC signaling, calcineurin and Hsp90 regulate resistance to drugs targeting the cell membrane in both *C. albicans* and *S. cerevisiae*, however, they regulate responses to echinocandins in *C. albicans* but not *S. cerevisiae* [27], [66], suggesting both conservation and divergence in circuitry governing fungal drug resistance.

The cellular circuitry linking PKC signaling, Hsp90, and calcineurin is complex with multiple levels of regulatory control. On one level is the connection between PKC signaling and calcineurin, which is divergent between the two species. In *S. cerevisiae*, inhibition of Pkc1 blocks calcineurin activation. The terminal MAPK Slt2 has been found to activate the Cch1-Mid1 high-affinity Ca^2+^ channel in response to endoplasmic reticulum stress, thereby enabling calcineurin activation [56]. However, we found that deletion of this channel had little impact on drug tolerance (Figure 4), implicating calcineurin regulation via a distinct mechanism. Since inhibition of PKC signaling does not affect calcineurin expression (Figure S5), Slt2 likely regulates calcineurin activation by an alternative mechanism such as through a distinct calcium channel. In *C. albicans*, *cch1Δ/cch1Δ* and *mid1Δ/mid1Δ* mutants share some but not all phenotypes with a calcineurin mutant [59]. Consistent with this, we found that the *cch1Δ/cch1Δ* and *mid1Δ/mid1Δ* mutants are almost as sensitive to fluconazole and fenpropimorph as a calcineurin mutant but only show an intermediate sensitivity to terbinafine (Figure 5). In *C. albicans*, inhibition of PKC signaling did not block calcineurin function (Figure 7). Our findings support a model in which Pkc1 and calcineurin independently regulate responses to ergosterol biosynthesis inhibitors in *C. albicans* via a common target that remains to be identified. On another level is the connection between Hsp90 and the terminal MAPK. In *S. cerevisiae*, Hsp90 interacts with activated Slt2 and enables activation of Slt2 targets including Rlm1 [64]. In *C. albicans*, Hsp90 stabilizes Mkc1 independent of its activation status (Figure 9). Notably, in *S. cerevisiae* Hsp90 also chaperones Pkc1 [72], though this has yet to be investigated in *C. albicans*. In contrast to the extensive Hsp90 network in *S. cerevisiae* [73], [74], we identify Mkc1 as the second Hsp90 client protein in *C. albicans*. Our work suggests that Hsp90 regulates responses crucial for survival of drug-induced membrane stress through PKC signaling in addition to the established role through calcineurin [24], [25], [27]. These stress responses are less important for resistance due to overexpression of multidrug transporters but are critical for basal tolerance as well as resistance acquired by other diverse mutations. Future experiments will address the relative contribution of calcineurin and PKC signaling via the MAPK cascade in Hsp90-mediated resistance acquired by diverse mechanisms.

Our results highlight the central importance of fungal stress response pathways in enabling survival in the hostile host environment. We demonstrate that while deletion of *PKC1* has little impact on growth *in vitro*, it drastically attenuates the capacity of *C. albicans* to proliferate *in vivo* and cause disease (Figure 10). While the attenuated virulence precludes studies to determine if compromising Pkc1 enhances the efficacy of antifungals *in vivo*, it provides compelling support for targeting fungal Pkc1 as a strategy to control fungal infections. The specific mechanism by which Pkc1 enables virulence has yet to be determined, however, it may operate in part via the downstream MAPK cascade given that *C. albicans* Mkc1 also contributes to virulence in a murine model [75]. While Mkc1 has little impact on susceptibility to oxidative-mediated killing by phagocytes [76], it is activated by physical contact and is required for invasive hyphal growth and normal biofilm development [77]. The mechanism by which Pkc1 influences virulence is distinct from calcineurin, which is required for *C. albicans* virulence and survival in the bloodstream [67], [68], [69]. While the calcineurin mutant is unable to survive in serum, the *pkc1Δ/pkc1Δ* mutant only exhibits an intermediate reduction in viability (Figure 10B), suggesting that Pkc1 regulates virulence via alternate targets. Notably, Pkc1 controls the expression of numerous virulence determinants in the fungal pathogen *Cryptococcus neoformans* [78], suggesting that Pkc1 governs virulence in phylogenetically diverse fungal species.

Our results suggest that targeting Pkc1 may provide a powerful strategy for the treatment of fungal infectious disease. *In vitro*, compromising PKC signaling renders laboratory strains and clinical isolates hypersensitive to drugs targeting ergosterol biosynthesis (Figures 1, 2, 3, and 8). These findings coupled with those established by others linking PKC signaling to tolerance of drugs targeting the cell wall [34], [35], [36], [37], suggest that compromising Pkc1 could have therapeutic benefits by enhancing the efficacy of the two most widely deployed classes of antifungals, the azoles and echinocandins. In a murine model of disseminated candidiasis, deletion of *PKC1* attenuates *C. albicans* virulence (Figure 10A), suggesting therapeutic benefit of simply compromising fungal Pkc1 in addition to the benefits of combinatorial therapeutic strategies. Notably, in mammalian cells disruption of PKC signaling impairs tumor progression and drug resistance such that PKC inhibitors have entered clinical trials for the treatment of several human cancers as single or combination therapy agents [79], [80]. The complexity of functions and interactions of mammalian PKC isoforms poses a challenge for the development of anti-cancer therapeutics and current efforts focus on enhancing specificity of action to target specific isoforms. While *C. albicans* and other fungal pathogens only have one PKC isoform, the therapeutic challenge will lie in achieving fungal selectivity. The successful development of Hsp90 and calcineurin as therapeutic targets for fungal disease faces similar challenges due to complications of inhibiting the function of these key cellular regulators in the host [66], [81], [82]. As a complement to identifying fungal selective pharmacological agents, elucidating the architecture of cellular circuitry governing stress responses, drug resistance, and virulence is poised to reveal promising therapeutic targets as key points of regulatory control that diverged between pathogen and host.

## Materials and Methods

### Ethics statement

All procedures were approved by the Institutional Animal Care and Use Committee (IACUC) at Duke University according to the guidelines of the Animal Welfare Act, The Institute of Laboratory Animal Resources Guide for the Care and Use of Laboratory Animals, and Public Health Service Policy.

### Strains and culture conditions

Archives of *C. albicans* and *S. cerevisiae* strains were maintained at −80°C in 25% glycerol. Strains were grown in either yeast peptone dextrose (YPD, 1% yeast extract, 2% bactopeptone, 2% glucose) or in synthetic defined medium (SD, 0.67% yeast nitrogen base, 2% glucose) and supplemented with amino acids or in RPMI medium 1640 (Gibco SKU#318000-089, 3.5% MOPS, 2% glucose, pH 7.0) supplemented with amino acids. 2% agar was added for solid media. Strains were transformed following standard protocols. Strains used in this study are listed in Table S1. Strain construction is described in Text S1.

### Plasmid construction

Recombinant DNA procedures were performed according to standard protocols. Plasmids used in this study are listed in Table S2. Plasmid construction is described in Text S1. Plasmids were sequenced to verify the absence of any nonsense mutations. Primers used in this study are listed in Table S3.

### Isolation and characterization of cercosporamide

A seed culture of the fungus, *Mycosphaerella* (*Cercosporidium*) *henningsii* (IMI 176827) grown on potato dextrose agar (PDA) for two weeks was used for inoculation. Mycelia were scraped out and mixed with 20 mL sterile water and filtered through a 100 µm filter. Absorbance of the spore suspension was measured and adjusted to 0.4. A 2 L Erlenmeyer flask containing 1 L of M-1-D medium [83] was inoculated with 10 mL of the spore suspension and incubated at 160 rpm and 28°C for four weeks. Mycelia were then separated from the supernatant by filtration through Whatman No. 1 filter paper and the filtrate was extracted with EtOAc (6×500 mL). The combined EtOAc extracts were washed with H_2_O (3×500 mL), dried over anhydrous Na_2_SO_4_ and evaporated under reduced pressure to yield a dark brown semi-solid (51.2 mg). A portion (50.0 mg) of the EtOAc extract was separated on preparative TLC (Merck, TLC silica gel 60 F_254_ precoated Aluminum sheets) using MeOH/Et_2_O (3∶97) as eluant affording crude cercosporamide (8.1 mg, *R_f_* 0.4). This was further purified by reversed-phase preparative TLC (Merck, TLC Silica gel 60 RP-18 F_254_ precoated Aluminium sheets) using H_2_O/CH_3_CN (3∶7) as eluant to give pure cercosporamide (4.5 mg, *R_f_* 0.5).

**Cercosporamide**: red crystals; mp 187–188°C (lit. [46] 188–189°C); APCIMS (+)-ve mode, m/z 331 [M+1]^+^; ^1^H and ^13^C NMR spectroscopic data were consistent with those reported in the literature [46]. The structure of cercosporamide is shown in Figure S9.

### Minimum inhibitory concentration and checkerboard assays

Antifungal tolerance and resistance were determined in flat bottom, 96-well microtiter plates (Sarstedt) using a modified broth microdilution protocol as described [25], [27]. Dimethyl sulfoxide (DMSO, Sigma Aldrich Co.) was the solvent for fenpropimorph (FN, Sigma Aldrich Co) and terbinafine (TB, Sigma Aldrich Co.); fluconazole (FL, Sequoia Research Products) and micafungin (MF, generously provided by Julia R. Köhler) were dissolved in sterile ddH_2_O. Geldanamycin (GdA, A.G. Scientific, Inc.) was used to inhibit Hsp90 at the indicated concentrations. Cyclosporin A (CsA, Calbiochem) was used to inhibit calcineurin at the indicated concentrations. Cercosporamide and staurosporine (STS, A.G. Scientific, Inc.) were used to inhibit protein kinase C at the indicated concentrations. DMSO was the solvent for GdA, CsA, STS, and cercosporamide.

Minimum inhibitory concentration (MIC) tests were set up in a total volume of 0.2 ml/well with 2-fold dilutions of FL, FN, TB and cercosporamide. FL gradients were from 256 µg/ml down to 0 with the following concentration steps in µg/ml: 256, 128, 64, 32, 16, 8, 4, 2, 1, 0.5, 0.25. FN gradients were from 25 µg/ml down to 0 with the following concentration steps in µg/ml: 25, 12.5, 6.25, 3.125, 1.5625, 0.78125, 0.390625, 0.1953125, 0.09765625, 0.04882813, 0.02441406. TB gradients were from 250 µg/ml with the following concentration steps in µg/ml: 250, 125, 62.5, 31.25, 15.625, 7.8125, 3.90625, 1.953125, 0.9765625, 0.48828125, 0.24414063. Cercosporamide gradients were from 100 µg/ml with the following concentration steps in µg/ml: 100, 50, 25, 12.5, 6.25, 3.125, 1.5625, 0.78125, 0.390625, 0.1953125, 0.09765625. Cell densities of overnight cultures were determined and dilutions were prepared such that ∼10^3^ cells were inoculated into each well. Plates were incubated in the dark at 30°C or 35°C for the period of time indicated in the figure legend, at which point plates were sealed with tape and re-suspended by agitation. Absorbance was determined at 600 nm using a spectrophotometer (Molecular Devices) and corrected for background from the corresponding medium. Each strain was tested in duplicate on at least 3 occasions. MIC data was quantitatively displayed with color using the program Java TreeView 1.1.1 (http://jtreeview.sourceforge.net).

Checkerboard assays were set up in a total volume of 0.2 ml/well with 2-fold dilutions of cyclosporin A across the x-axis of the plate and 2-fold dilutions of STS across the y-axis of the plate. STS gradients were from 0.5 µg/ml to 0 in the following concentrations steps in µg/ml: 0.5, 0.25, 0.125, 0.0625, 0.03125, 0.015625, 0.0078125. CsA gradients were from 48 µg/ml down to 0 in the following concentration steps in µM: 48, 24, 12, 6, 3, 1.5, 0.75, 0.375, 0.1875, 0.09375, 0.046875. Plates were inoculated and growth was measured as with MIC tests. To test for synergy, the fractional inhibitory concentration (FIC) was calculated as follows: [(MIC_80_ of drug A in combination)/(MIC_80_ of drug A alone)] + [(MIC_80_ of drug B in combination)/(MIC_80_ of drug B alone)]. Values of ≤0.5 indicate synergy, those of >0.5 but <2 indicate no interaction and those ≥2 indicate antagonism.

### Spotting assays

Strains were grown overnight to saturation in indicated media and cell concentrations were determined based on cell counts using a hemacytometer (Hausser Scientific). Five-fold serial dilutions of cell suspensions starting at indicated concentrations (10^5^ or 10^7^cells/ml) were performed in sterile ddH_2_O or sterile phosphate buffered saline. Cell suspensions were spotted onto indicated media using a spotter (Frogger, V&P Scientific, Inc). Plates were photographed after 3 days in the dark at indicated temperature.

### Cidality assay

For *S. cerevisiae*, MIC assays with two-fold dilutions of FL, FN, or TB were performed in SD as described above. For FL the gradients were from 256 µg/ml down to 0 with the following concentration steps in µg/ml: 256, 128, 64, 32, 16. FN gradients were from 100 µg/ml down to 0 with the following concentration steps in µg/ml: 100, 50, 25, 12.5, 6.25. TB gradients were from 250 µg/ml with the following concentration steps in µg/ml: 250, 125, 62.5, 31.25, 15.62. Plates were incubated for two days at 35°C. Cells from the MIC assay were spotted onto solid YPD medium and incubated at 30°C for two days before they were photographed.

For *C. albicans*, MIC assays with FL, FN, or TB were performed in YPD as described above with the following modification; four-fold dilutions of each drug were tested. For FL the gradients were from 256 µg/ml down to 0 with the following concentration steps in µg/ml: 256, 64, 16, 4, 1. FN gradients were from 25 µg/ml down to 0 with the following concentration steps in µg/ml: 25, 6.25, 1.5625, 0.390625, 0.09765625. TB gradients were from 250 µg/ml with the following concentration steps in µg/ml: 250, 62.5, 15.625, 3.90625, 0.9765625. Plates were incubated for two days at 35°C. Cells from the MIC assay were spotted onto solid YPD medium and incubated at 30°C for two days before they were photographed.

### β-galactosidase assays

*S. cerevisiae* cultures were grown overnight at 25°C in SD medium supplemented for auxotrophies. Cells were diluted to OD_600_ of 0.05 and were either left untreated or were treated with FL (16 µg/ml), FN (1 µg/ml), or TB (25 µg/ml) for 24 hours at 25°C. When STS was used as an inhibitor in the assay, cultures were grown overnight in SD at 25°C and diluted to OD_600_ of 0.05 in SD with or without STS (2.5 µg/ml) for 24 hours at 25°C. Cells were then diluted to OD_600_ of 0.05 in SD with or without STS and with or without FL (32 µg/ml) for an additional 24 hours at 25°C. Cells were harvested, washed, protein was extracted, and protein concentrations were determined by Bradford analysis as described [27]. Protein samples were diluted to the same concentration and β-galactosidase activity was measured using the substrate ONPG (*O*-nitrophenyl-β-D-galactopyranosidase, Sigma Aldrich Co.) as described [27]. β-galactosidase activity is given in units of nanomoles ONPG converted per minute per milligram of protein. Statistical significance was evaluated using GraphPad Prism 4.0.

### Immune blot analysis

For the Mkc1 activation assay, yeast cultures were grown overnight in YPD at 30°C. In the morning, cells were diluted to OD_600_ of 0.2 in 50 mL YPD and were grown to mid-log (∼3 hours) at 30°C and then cultures were split into 5×10 mL cultures and were either left untreated or were treated with FL (8 µg/mL), FN (1 µg/mL), MF (30 ng/mL), or TB (25 µg/ml) for 2 hours at 30°C. Cells were harvested by centrifugation at 1308×g for 10 minutes at 4°C and were washed with sterile cold phosphate buffered saline (PBS). Cell pellets were resuspended in lysis buffer containing 50 mM HEPES pH 7.4, 150 mM NaCl, 5 mM EDTA, 1%Triton ×100, 50 mM NaF, 10 mM Na_3_VO_4_, 1 mM PMSF, and protease inhibitor cocktail (complete, EDTA-free tablet, Roche Diagnostics).

For the Mkc1 destabilization assay, cultures were grown overnight in YPD at 30°C. In the morning, cells were diluted to OD_600_ of 0.2 in 10 mL YPD with or without doxycycline (20 µg/mL; BD Biosciences) and left at 30°C for 24 hours. Cells were diluted once again to OD_600_ of 0.2 in the same treatment conditions as overnight and were grown at 30°C until mid-log phase (∼4 hours). Doxycycline reduces the growth rate of strains with the repressible promoter driving expression of the only *HSP90* allele but does not affect stationary phase cell density [65]. Cells were then treated with 50 µg/mL TB for 3 hours at 30°C to elicit phosphorylation of Mkc1. Cells were harvested after TB treatment at 1308×g at 4°C and washed with ice-cold ddH_2_O. Cell pellets were flash frozen in liquid N_2_, resuspended in lysis buffer (50 mM HEPES pH 7.4, 150 mM NaCl, 5 mM EDTA, 1% Triton ×100, 100 mM NaF, 20 mM Na_3_VO_4_, 1 mM PMSF and protease inhibitor cocktail complete, EDTA-free tablet, Roche Diagnostics).

Cells suspended in lysis buffer were mechanically disrupted by adding acid-washed glass beads and bead beating for 1 minute for six cycles with 1 minute on ice between each cycle. Protein concentrations were determined by Bradford analysis. Protein samples were mixed with one-sixth volume of 6× sample buffer containing 0.35 M Tris-HCl, 10% (w/w) SDS, 36% glycerol, 5% β-mercaptoethanol, and 0.012% bromophenol blue for SDS-PAGE. Samples were boiled for 5 minutes and then separated by 10% SDS-PAGE. Protein was electrotransferred to PVDF membrane (Bio-Rad Laboratories, Inc.) and blocked with 5% skimmed milk in PBS with 0.1% tween or 5% bovine serum albumin in phosphate buffered saline with 0.1% tween. Blots were hybridized with antibodies against CaHsp90 (1∶10000 dilution, generously provided by Brian Larsen, [84]), histone H3 (1∶3000 dilution; Abcam ab1791), His_6_ (1∶10, P5A11, generously provided by Elizabeth Wayner) and phospho-p44/42 MAPK (Thr202/Tyr204) (1∶2000, Cell Signaling).

### Quantitative reverse transcription-PCR (qRT-PCR)

To monitor gene expression changes in response to FL treatment in *S. cerevisiae*, cells were grown overnight in SD supplemented for auxotrophies at 30°C. Cells were diluted to OD_600_ of 0.1 in SD and grown for 2 hours in duplicate at 25°C. After 2 hours of growth 16 µg/mL FL was added to one of the two duplicate cultures and left to grow for an additional 4 hours at 25°C. Cell pellets were frozen at −80°C immediately.

To monitor gene expression changes in response to FL treatment in *C. albicans*, cells were grown overnight in YPD at 30°C. Cells were diluted to OD_600_ of 0.1 in YPD and grown for 2 hours in duplicate at 35°C. After 2 hours of growth 16 µg/mL FL was added to one of the two duplicate cultures and left to grow for an additional 4 hours at 35°C. Cell pellets were frozen at −80°C immediately.

To monitor *MKC1* transcript levels in response to decreased levels of Hsp90, cultures were grown overnight in YPD at 30°C. In the morning, cells were diluted to OD_600_ of 0.2 in 10 mL YPD with or without 20 µg/mL doxycycline (BD Biosciences) and left at 30°C for 24 hours. The next morning, cells were diluted once again to OD_600_ of 0.2 in the same treatment conditions and were grown at 30°C until mid-log phase (∼4 hours). Cell pellets were collected and immediately frozen at −80°C.

RNA was isolated using the QIAGEN RNeasy kit and RNAase-free DNase (QIAGEN), and cDNA synthesis was performed using the AffinityScript cDNA synthesis kit (Stratagene). PCR was performed using SYBR Green JumpStart Taq ReadyMix (Sigma-Aldrich Co.) with the following cycling conditions: 94°C for 2 minutes, 94°C for 15 seconds, 60°C for 1 minute, 72°C for 1 minute, for 30 or 40 cycles. All reactions were performed in triplicate, using primers for the following genes: *CaGPD1* (oLC752/753), *CaHSP90* (oLC754/755), *ScACT1* (oLC1015/1016), *ScCNA1* (oLC1286/1287), *ScCNA2* (oLC1288/1289), *ScCNB1*(oLC1290/1291), *CaCNB1* (oLC1292/1293), *CaCNA1* (oLC1294/1295), *ScCRZ1* (oLC1328/1329), *CaCRZ1*(oLC1330/1331), *CaMKC1*(oLC1332/1333), *CaPLC3*(oLC1432/1433), and *CaUTR2*(oLC1434/1435). Data were analyzed using iQ5 Optical System Software Version 2.0 (Bio-Rad Laboratories, Inc.). Statistical significance was evaluated using GraphPad Prism 4.0.

### Murine model of *C. albicans* infection

Inoculum was prepared as described [20], [27], [65]. Cultures were started from frozen stocks onto Sabouraud dextrose agar plates and incubated at 35°C for 48 hours. Colonies were suspended in sterile pH 7.4 PBS, centrifuged at 324×g for 5 minutes, washed with sterile PBS one time and diluted to the desired concentration as verified by counting on a Neubauer hematocytometer as well as by serial dilution and culture. Male CD1 mice (Charles River Laboratories, Wilmington, MA) age 8 weeks (weight 30–34 g) were infected via the tail vein with 100 µL of a 1×10^6^ CFU/mL suspension of the wild type strain (CaLC239, 1×10^5^ CFU per mouse, *n* = 9 mice), an inoculum previously determined to produce morbidity but not mortality when using *C. albicans* strain SC5314 at 4 days following tail vein injection (Zaas *et al.* unpublished data). We observed discordance between cell counts and CFU measurements for the *pk1cΔ/pkc1Δ* mutant, such that CFU values were ∼50% lower than expected; thus, inocula for the *pk1cΔ/pkc1Δ* mutant were prepared at higher concentrations based on cell counts and the effective concentrations in CFUs were confirmed by dilution plating. For infection with the *pk1cΔ/pkc1Δ* mutant, we used an inoculum equivalent to that for the wild type (1×10^5^ CFU, *n* = 8 mice) as well a 10-fold and 100-fold increase in inoculum (1×10^6^, *n* = 11 mice and 1×10^7^ CFU, *n* = 8 mice). Mice were observed three times daily for signs of illness and weighed daily. At day 4 following injection, mice were sacrificed using CO_2_ asphyxiation and the left kidney was removed aseptically, placed in sterile PBS, homogenized using a FastPrep 120 (QBiogene) using 0.5 mm zirconium beads (Biospec, Inc.) for 1 minute and serial dilutions plated for determination of kidney fungal burden. The CFU values in kidneys were expressed as CFU/g of tissue and log-transformed. Statistical significance was evaluated using GraphPad Prism 4.0.

### Accession numbers for genes and proteins mentioned in text (NCBI Entrez gene ID number)

*S. cerevisiae: PKC1* (852169); *HSC82* (855224); *HSP82* (855836); *CNA1* (851153); *CNA2* (854946); *CNB1* (853644); *ERG11* (856398); *ERG3* (850745); *RHO1* (856294); *BCK1* (853350); *MKK1* (854406); *MKK2* (855963); *SLT2* (856425); *SWI4* (856847); *SWI6* (850879); *ERG2* (855242); *ERG24* (855441); *ERG1* (853086); *RLM1* (856016); *CCH1* (853131); *MID1* (855425); *CRZ1* (855704); *PDR5* (854324); *PDR1* (852871); *PDR3* (852278); *ACT1* (850504).

*C. albicans PKC1* (3635298); *HSP90* (3637507); *CNA1* (3639406); *CNB1* (3636463); *MKC1* (3639710); *ERG11* (3641571); *ERG3* (3644776); *RHO1* (3642564); *BCK1* (3641434); *MKK2* (3645580); *MDR1* (3639260); *ERG2* (3639416); *ERG24* (3648198); *ERG1* (3646509); *CEK1* (3642789); *CEK2* (3642459); *RLM1* (3635703); *SWI4* (3645507); *SWI6* (3634957); *CCH1* (3639950); *MID1* (3647441); *CRZ1* (3641722); *PLC3* (3635941); *UTR2* (3636747); *CDR1* (3635385); *GPD1* (3643986).

## Supporting Information

Figure S1 Pkc1-MAPK signaling enables tolerance to ergosterol biosynthesis inhibitors in *S. cerevisiae*. (**A**) At the permissive temperature, the *pkc1-ts* mutant and the wild-type (WT) strain (BY4741) have comparable tolerance to all three ergosterol biosynthesis inhibitors tested. Assays were performed in synthetic defined (SD) medium at 35°C. Data was analyzed after 48 hours as in Figure 1A. (**B**) Genetic compromise of Pkc1 function reduces ergosterol biosynthesis inhibitor tolerance on solid SD medium. Drug tolerance of a WT strain (BY4741) and a derivative (*pkc1-ts*) with a temperature sensitive *PKC1* allele spotted in fivefold dilutions (from 1×10^7^ cells/ml) onto plates with no antifungal (-) or with a fixed concentration of fluconazole (FL, 16 µg/mL), terbinafine (TB, 30 µg/mL), or fenpropimorph (FN, 0.5 µg/mL), as indicated. Plates were incubated at 30°C or 35°C and were photographed after 72 hours at the indicated temperatures. (**C**) Deletion of components of the MAPK cascade, *BCK1* and *SLT2*, reduces ergosterol biosynthesis inhibitor tolerance on solid SD medium. Assays were performed as indicated in (A) and plates were incubated 35°C and photographed after 72 hours.
(2.41 MB TIF)

Figure S2 Restoring a wild-type *PKC1* allele restores basal tolerance to ergosterol biosynthesis inhibitors in *C. albicans*. (**A**) Homozygous deletion of *PKC1* reduces ergosterol biosynthesis inhibitor tolerance on solid YPD medium and restoring a wild-type *PKC1* allele restores tolerance. Cells were spotted in fivefold dilutions (from 1×10^7^ cells/ml for *pkc1Δ/pkc1Δ* and from 1×10^5^ cells/ml for other strains) onto plates with no antifungal (-) or with a fixed concentration of fluconazole (FL, 4µg/mL), terbinafine (TB, 2.5µg/mL), or fenpropimorph (FN, 0.25µg/mL). Plates were photographed after 72 hours growth at 35°C. (**B**) Homozygous deletion of *PKC1* confers hypersensitivity to all three ergosterol biosynthesis inhibitors tested in MIC assays; restoring a wild-type *PKC1* allele under the control of the native promoter to the native locus restores basal tolerance. Assays were performed in YPD medium at 30°C with strains derived from the WT SN95. Data was analyzed after 72 hours growth as in Figure 1A. (**C**) Homozygous deletion of *PKC1* renders the ergosterol biosynthesis inhibitors fungicidal against *C. albicans* and restoring a wild-type *PKC1* allele restores the fungistatic activity. MIC assays with four-fold dilutions of FL, FN, and TB were performed in YPD and incubated for 48 hours at 35°C. Cells from the MIC assays were spotted onto YPD medium and incubated at 30°C for 48 hours before plates were photographed.
(1.85 MB TIF)

Figure S3 Involvement of the *C. albicans* MAPK cascade in responses to ergosterol biosynthesis inhibitors at 30°C but not 35°C. (**A**) Exposure of *C. albicans* with ergosterol biosynthesis inhibitors activates the MAPK cascade leading to the accumulation of phosphorylated Mkc1. One allele of *MKC1* was C-terminally 6xHis–FLAG tagged in this strain. Cells were grown to mid-log before being treated for 2 hours at 30°C as follows: untreated (-); FN, 1 µg/mL; FL, 8 µg/mL; TB, 25 µg/mL; or MF, 30 ng/mL. Total protein resolved by SDS-PAGE was blotted with α-His_6_ to monitor total Mkc1 levels and α-phospho p44/42 MAPK to monitor dually phosphorylated Mkc1 levels. (**B**) Deletion of *BCK1* and *MKC1* does not increase sensitivity to fluconazole (FL), fenpropimorph (FN), or terbinafine (TB) in MIC assays performed in YPD medium at 35°C. Data was analyzed after 72 hours as in Figure 1A.
(0.31 MB TIF)

Figure S4 Pkc1 enables tolerance to ergosterol biosynthesis inhibitors in *S. cerevisiae* via the MAPK cascade at 30°C. Deletion of components of the MAPK cascade in the BY4741 background confers hypersensitivity to ergosterol biosynthesis inhibitors in MIC assays conducted in SD at 30°C. Data was analyzed after 48 hours as in Figure 1A.
(0.18 MB TIF)

Figure S5 Genetic perturbation of PKC signaling in *S. cerevisiae* does not compromise expression of calcineurin subunits or *CRZ1*. Deletion of *SLT2* does not reduce expression of calcineurin subunits or *CRZ1* in the presence or absence of ergosterol biosynthesis inhibitors. Transcript levels of the genes encoding the catalytic subunit (*CNA1* and *CNA2*) and the regulatory subunit (*CNB1*) of calcineurin and *CRZ1* were measured by quantitative RT-PCR after growth in SD at 25°C for 6 hours without any antifungal (U) or for two hours untreated followed by 4 hours with 16 µg/mL fluconazole (FL), as indicated. Transcripts were normalized to *ACT1*. Levels are expressed relative to the untreated wild-type samples, which were set to 1. Data are means ± SD for triplicate samples.
(0.46 MB TIF)

Figure S6 Inhibition of Pkc1 signaling phenocopies inhibition of Hsp90 reducing azole resistance of specific mutants. Azole resistance of matched sets of *C. albicans* clinical isolates (CaCi) taken from HIV-infected patients early (E) and late (L) during the course of fluconazole (FL) treatment is tested in MIC assays. Assays were conducted in YPD medium with no inhibitor (-), with the Hsp90 inhibitor geldanamycin (5 µM), or with the Pkc1 inhibitor staurosporine (0.5 µg/ml). Mutations implicated in azole resistance for each isolate are indicated. Data was analyzed after growth for 48 hours at 30°C as in Figure 1A.
(0.26 MB TIF)

Figure S7 The C-terminal 6xHis-FLAG epitope tag does not disrupt functionality of *C. albicans* Mkc1. When *MKC1-6xHis-FLAG* is expressed as the sole copy *MKC1*, it is sufficient to confer WT tolerance to the ergosterol biosynthesis inhibitors in MIC assays. Assays were performed in YPD and growth was measured after 48 hours at 30°C. Data was analyzed and plotted as in Figure 1A.
(0.26 MB TIF)

Figure S8 Genetic depletion of Hsp90 does not affect *MKC1* transcript levels. In strains where the sole allele of *HSP90* is under the control of a tetracycline repressible promoter (*tetO*), transcription of *HSP90* can be repressed by tetracycline or the analog doxycycline. Cells were grown in YPD with or without doxycycline (20 µg/ml) and *MKC1* transcript levels were measured by quantitative RT-PCR. Transcripts were normalized to *GPD1*. Levels are expressed relative to the untreated wild-type samples, which were set to 1. Data are means ± SD for triplicate samples.
(0.40 MB TIF)

Figure S9 Structure of cercosporamide. The structure of cercoposamide was made using ChemDraw Pro (CyberChem, Inc.).
(0.05 MB TIF)

Table S1 Strains used in this study.
(0.17 MB DOC)

Table S2 Plasmids used in this study
(0.03 MB DOC)

Table S3 Primers used in this study.
(0.04 MB DOC)

Text S1 Supporting Materials and Methods.
(0.06 MB DOC)
