# Supplementary figures and images for: PKC Signaling Regulates Drug Resistance of the Fungal Pathogen Candida albicans via Circuitry Comprised of Mkc1, Calcineurin, and Hsp90
Source: PLoS Pathog. 2010 Aug 26;6(8):e1001069. doi: 10.1371/journal.ppat.1001069 (PMC2928802; doi:10.1371/journal.ppat.1001069)

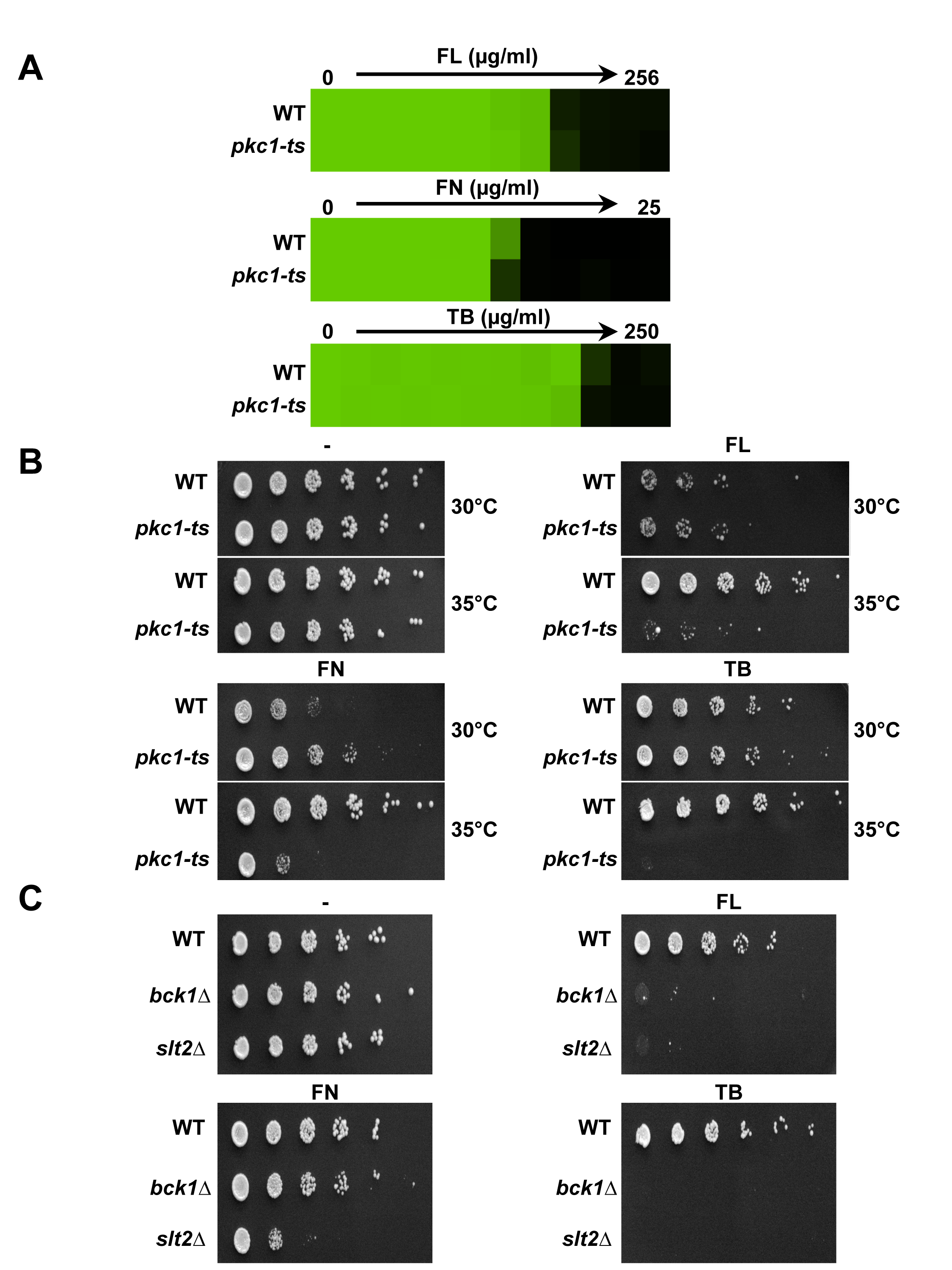

Supplement: Figure S1 — Pkc1-MAPK signaling enables tolerance to ergosterol biosynthesis inhibitors in S. cerevisiae. (A) At the permissive temperature, the pkc1-ts mutant and the wild-type (WT) strain (BY4741) have comparable tolerance to all three ergosterol biosynthesis inhibitors tested. Assays were performed in synthetic defined (SD) medium at 35°C. Data was analyzed after 48 hours as in Figure 1A. (B) Genetic compromise of Pkc1 function reduces ergosterol biosynthesis inhibitor tolerance on solid SD medium. Drug tolerance of a WT strain (BY4741) and a derivative (pkc1-ts) with a temperature sensitive PKC1 allele spotted in fivefold dilutions (from 1×10^7^ cells/ml) onto plates with no antifungal (-) or with a fixed concentration of fluconazole (FL, 16 µg/mL), terbinafine (TB, 30 µg/mL), or fenpropimorph (FN, 0.5 µg/mL), as indicated. Plates were incubated at 30°C or 35°C and were photographed after 72 hours at the indicated temperatures. (C) Deletion of components of the MAPK cascade, BCK1 and SLT2, reduces ergosterol biosynthesis inhibitor tolerance on solid SD medium. Assays were performed as indicated in (A) and plates were incubated 35°C and photographed after 72 hours. (2.41 MB TIF) [file ppat.1001069.s001.tif]

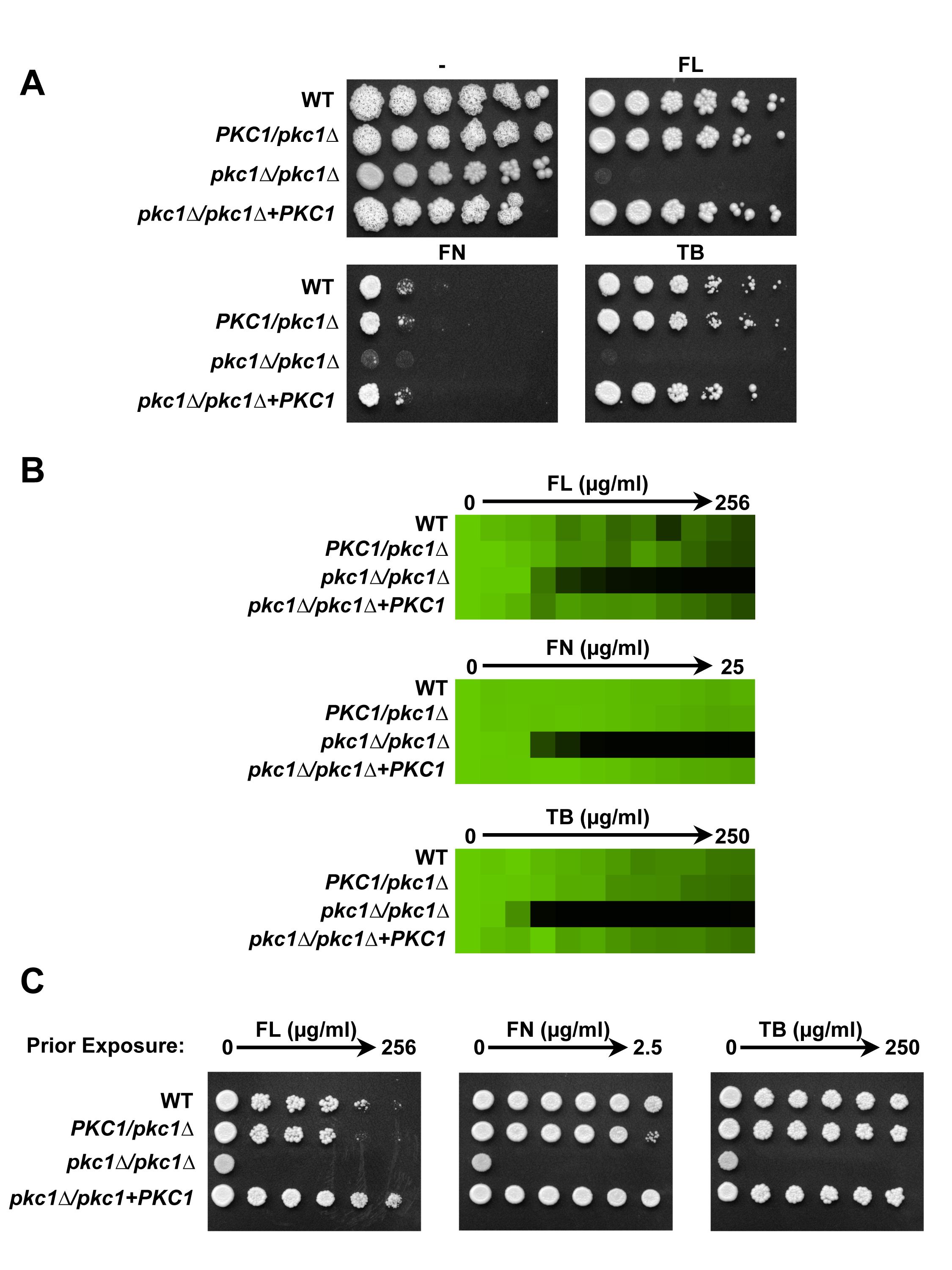

Supplement: Figure S2 — Restoring a wild-type PKC1 allele restores basal tolerance to ergosterol biosynthesis inhibitors in C. albicans. (A) Homozygous deletion of PKC1 reduces ergosterol biosynthesis inhibitor tolerance on solid YPD medium and restoring a wild-type PKC1 allele restores tolerance. Cells were spotted in fivefold dilutions (from 1×10^7^ cells/ml for pkc1Δ/pkc1Δ and from 1×10^5^ cells/ml for other strains) onto plates with no antifungal (-) or with a fixed concentration of fluconazole (FL, 4µg/mL), terbinafine (TB, 2.5µg/mL), or fenpropimorph (FN, 0.25µg/mL). Plates were photographed after 72 hours growth at 35°C. (B) Homozygous deletion of PKC1 confers hypersensitivity to all three ergosterol biosynthesis inhibitors tested in MIC assays; restoring a wild-type PKC1 allele under the control of the native promoter to the native locus restores basal tolerance. Assays were performed in YPD medium at 30°C with strains derived from the WT SN95. Data was analyzed after 72 hours growth as in Figure 1A. (C) Homozygous deletion of PKC1 renders the ergosterol biosynthesis inhibitors fungicidal against C. albicans and restoring a wild-type PKC1 allele restores the fungistatic activity. MIC assays with four-fold dilutions of FL, FN, and TB were performed in YPD and incubated for 48 hours at 35°C. Cells from the MIC assays were spotted onto YPD medium and incubated at 30°C for 48 hours before plates were photographed. (1.85 MB TIF) [file ppat.1001069.s002.tif]

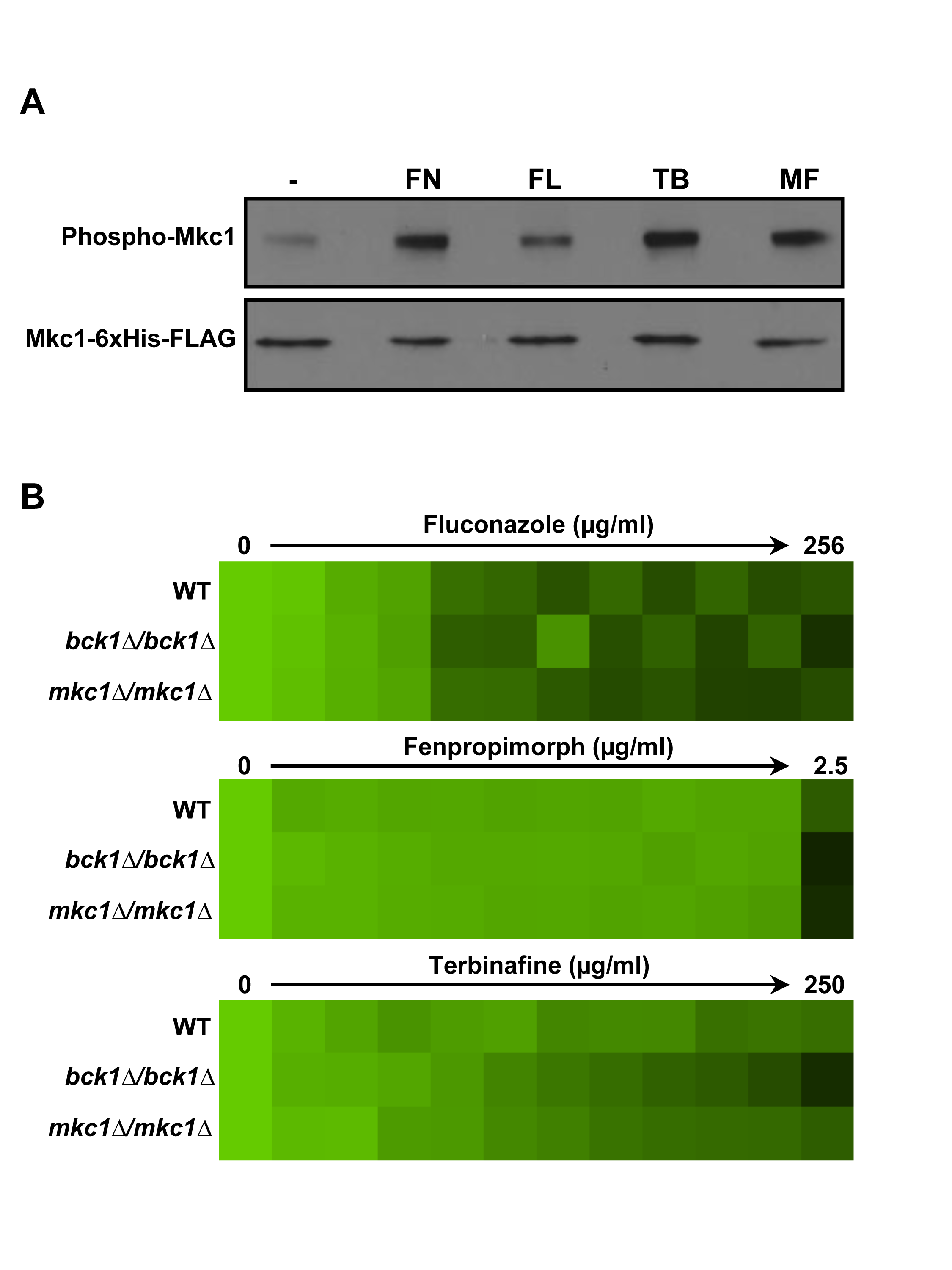

Supplement: Figure S3 — Involvement of the C. albicans MAPK cascade in responses to ergosterol biosynthesis inhibitors at 30°C but not 35°C. (A) Exposure of C. albicans with ergosterol biosynthesis inhibitors activates the MAPK cascade leading to the accumulation of phosphorylated Mkc1. One allele of MKC1 was C-terminally 6xHis–FLAG tagged in this strain. Cells were grown to mid-log before being treated for 2 hours at 30°C as follows: untreated (-); FN, 1 µg/mL; FL, 8 µg/mL; TB, 25 µg/mL; or MF, 30 ng/mL. Total protein resolved by SDS-PAGE was blotted with α-His6 to monitor total Mkc1 levels and α-phospho p44/42 MAPK to monitor dually phosphorylated Mkc1 levels. (B) Deletion of BCK1 and MKC1 does not increase sensitivity to fluconazole (FL), fenpropimorph (FN), or terbinafine (TB) in MIC assays performed in YPD medium at 35°C. Data was analyzed after 72 hours as in Figure 1A. (0.31 MB TIF) [file ppat.1001069.s003.tif]

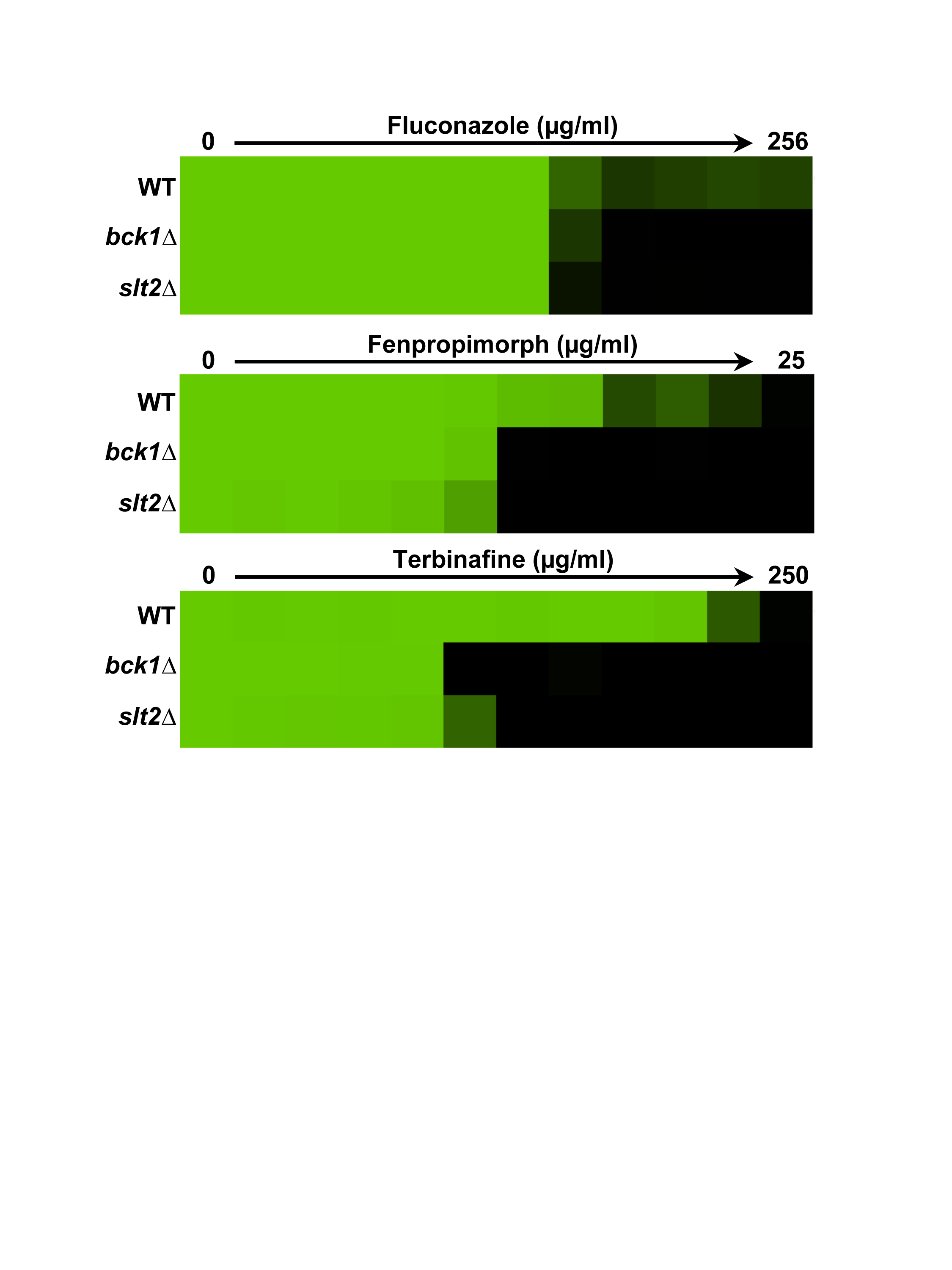

Supplement: Figure S4 — Pkc1 enables tolerance to ergosterol biosynthesis inhibitors in S. cerevisiae via the MAPK cascade at 30°C. Deletion of components of the MAPK cascade in the BY4741 background confers hypersensitivity to ergosterol biosynthesis inhibitors in MIC assays conducted in SD at 30°C. Data was analyzed after 48 hours as in Figure 1A. (0.18 MB TIF) [file ppat.1001069.s004.tif]

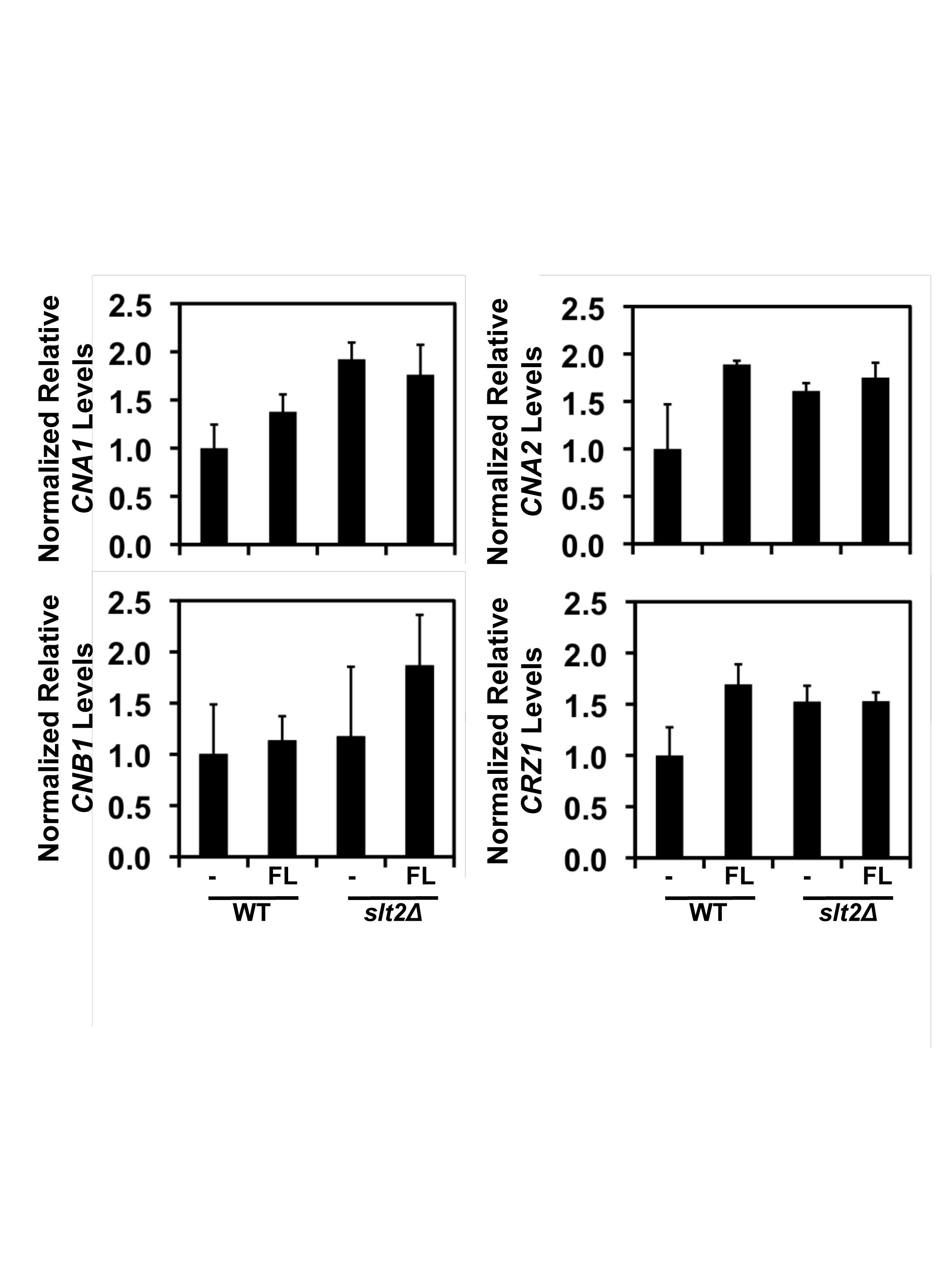

Supplement: Figure S5 — Genetic perturbation of PKC signaling in S. cerevisiae does not compromise expression of calcineurin subunits or CRZ1. Deletion of SLT2 does not reduce expression of calcineurin subunits or CRZ1 in the presence or absence of ergosterol biosynthesis inhibitors. Transcript levels of the genes encoding the catalytic subunit (CNA1 and CNA2) and the regulatory subunit (CNB1) of calcineurin and CRZ1 were measured by quantitative RT-PCR after growth in SD at 25°C for 6 hours without any antifungal (U) or for two hours untreated followed by 4 hours with 16 µg/mL fluconazole (FL), as indicated. Transcripts were normalized to ACT1. Levels are expressed relative to the untreated wild-type samples, which were set to 1. Data are means ± SD for triplicate samples. (0.46 MB TIF) [file ppat.1001069.s005.tif]

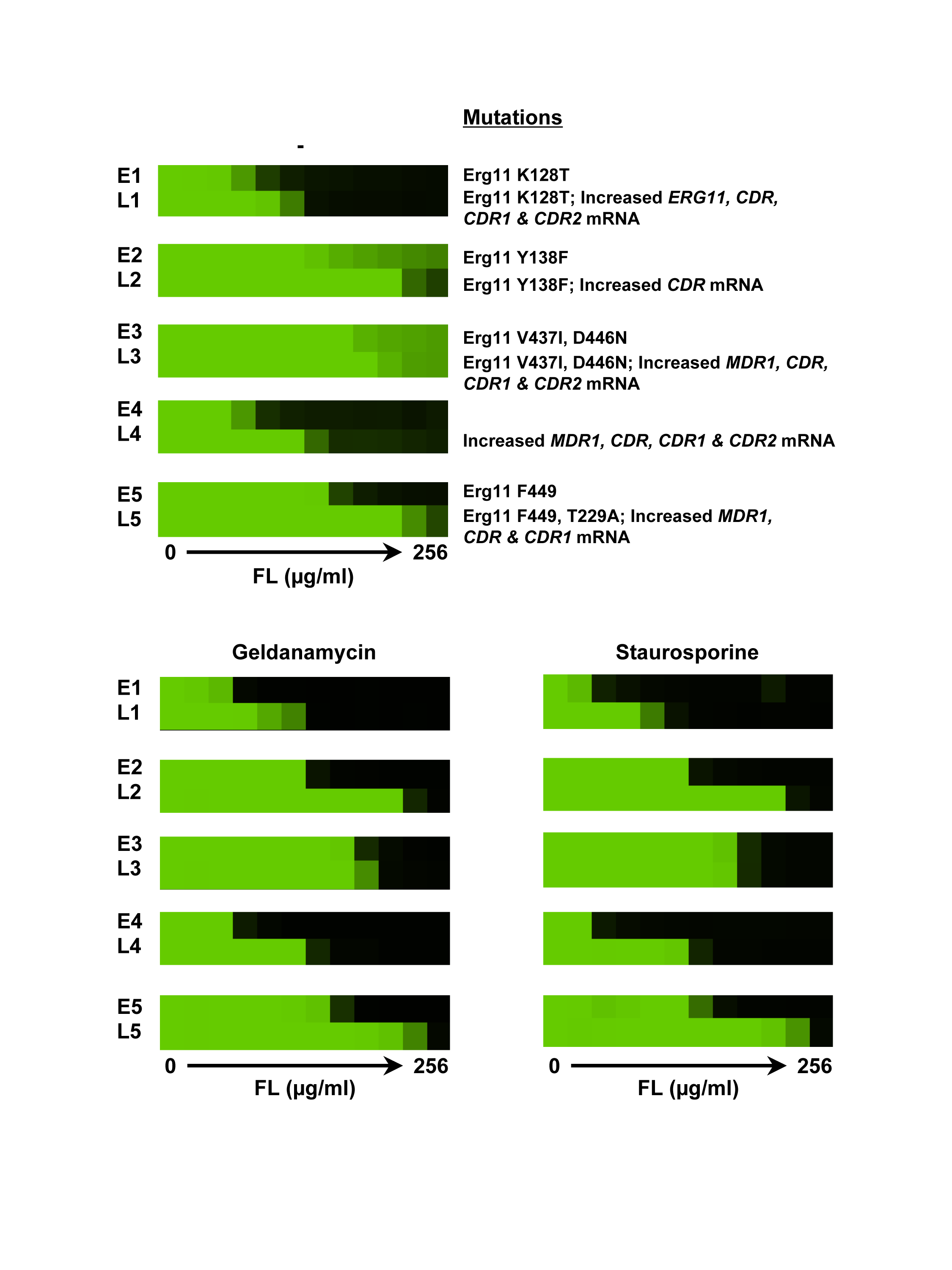

Supplement: Figure S6 — Inhibition of Pkc1 signaling phenocopies inhibition of Hsp90 reducing azole resistance of specific mutants. Azole resistance of matched sets of C. albicans clinical isolates (CaCi) taken from HIV-infected patients early (E) and late (L) during the course of fluconazole (FL) treatment is tested in MIC assays. Assays were conducted in YPD medium with no inhibitor (-), with the Hsp90 inhibitor geldanamycin (5 µM), or with the Pkc1 inhibitor staurosporine (0.5 µg/ml). Mutations implicated in azole resistance for each isolate are indicated. Data was analyzed after growth for 48 hours at 30°C as in Figure 1A. (0.26 MB TIF) [file ppat.1001069.s006.tif]

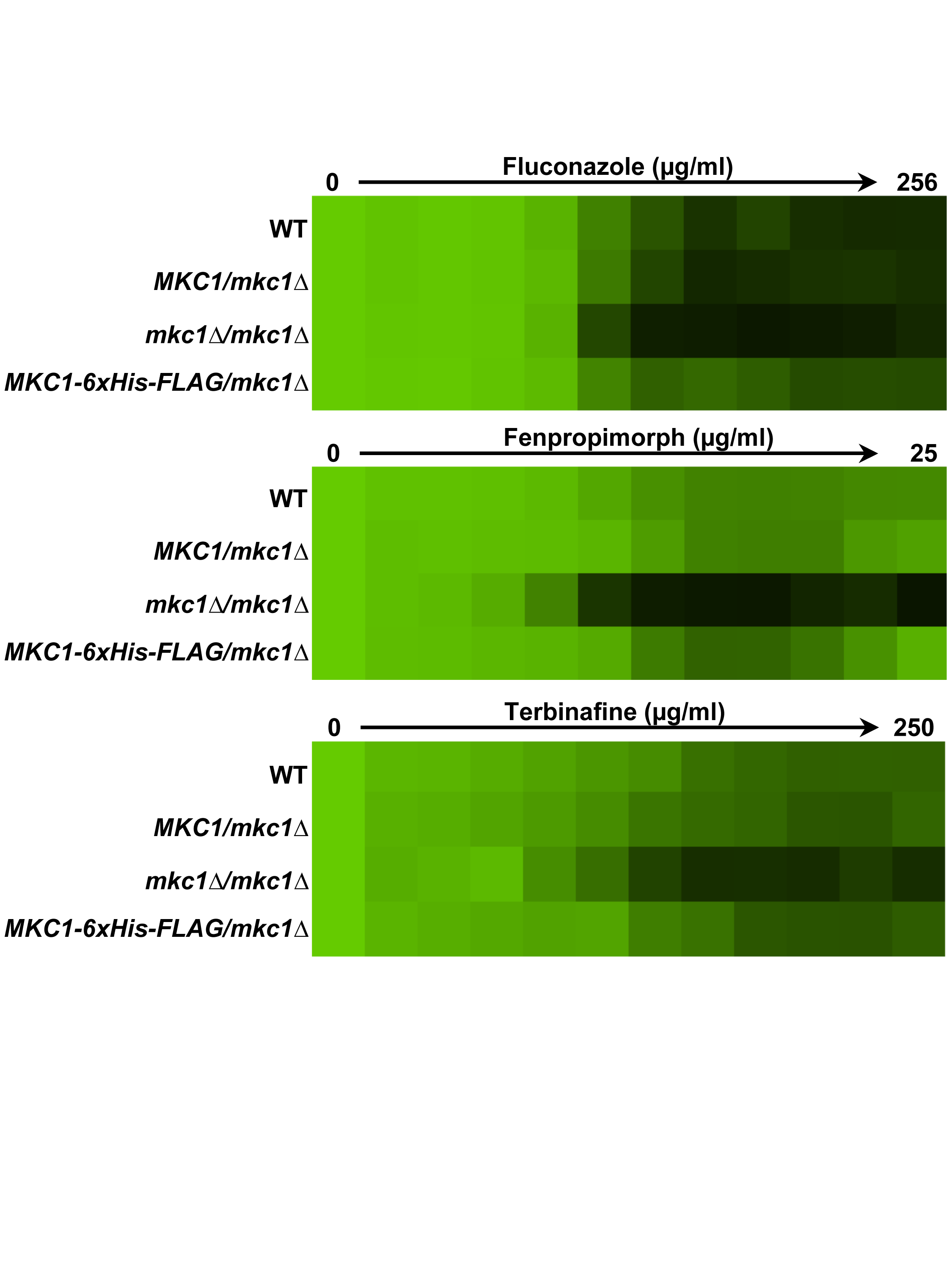

Supplement: Figure S7 — The C-terminal 6xHis-FLAG epitope tag does not disrupt functionality of C. albicans Mkc1. When MKC1-6xHis-FLAG is expressed as the sole copy MKC1, it is sufficient to confer WT tolerance to the ergosterol biosynthesis inhibitors in MIC assays. Assays were performed in YPD and growth was measured after 48 hours at 30°C. Data was analyzed and plotted as in Figure 1A. (0.26 MB TIF) [file ppat.1001069.s007.tif]

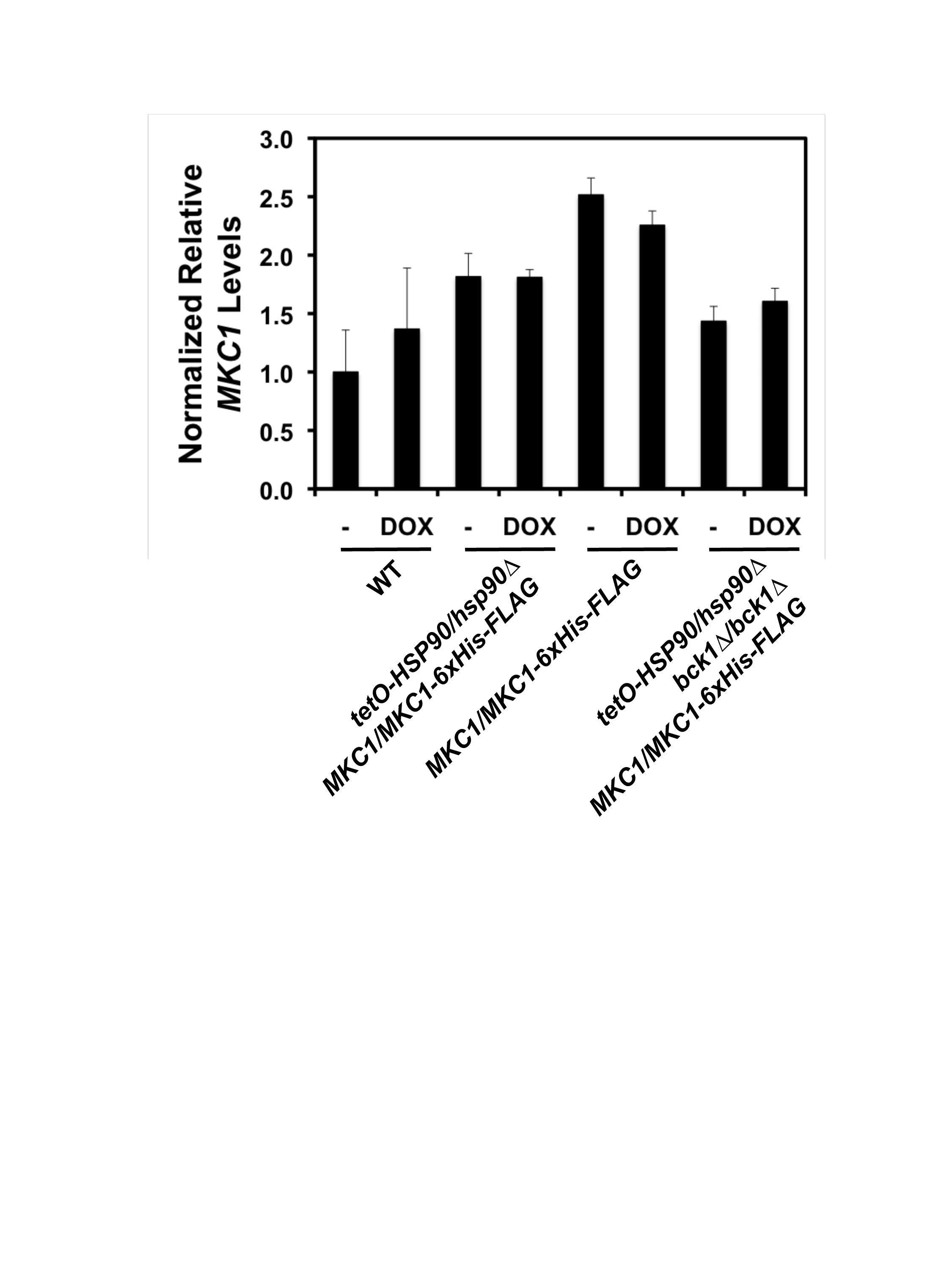

Supplement: Figure S8 — Genetic depletion of Hsp90 does not affect MKC1 transcript levels. In strains where the sole allele of HSP90 is under the control of a tetracycline repressible promoter (tetO), transcription of HSP90 can be repressed by tetracycline or the analog doxycycline. Cells were grown in YPD with or without doxycycline (20 µg/ml) and MKC1 transcript levels were measured by quantitative RT-PCR. Transcripts were normalized to GPD1. Levels are expressed relative to the untreated wild-type samples, which were set to 1. Data are means ± SD for triplicate samples. (0.40 MB TIF) [file ppat.1001069.s008.tif]

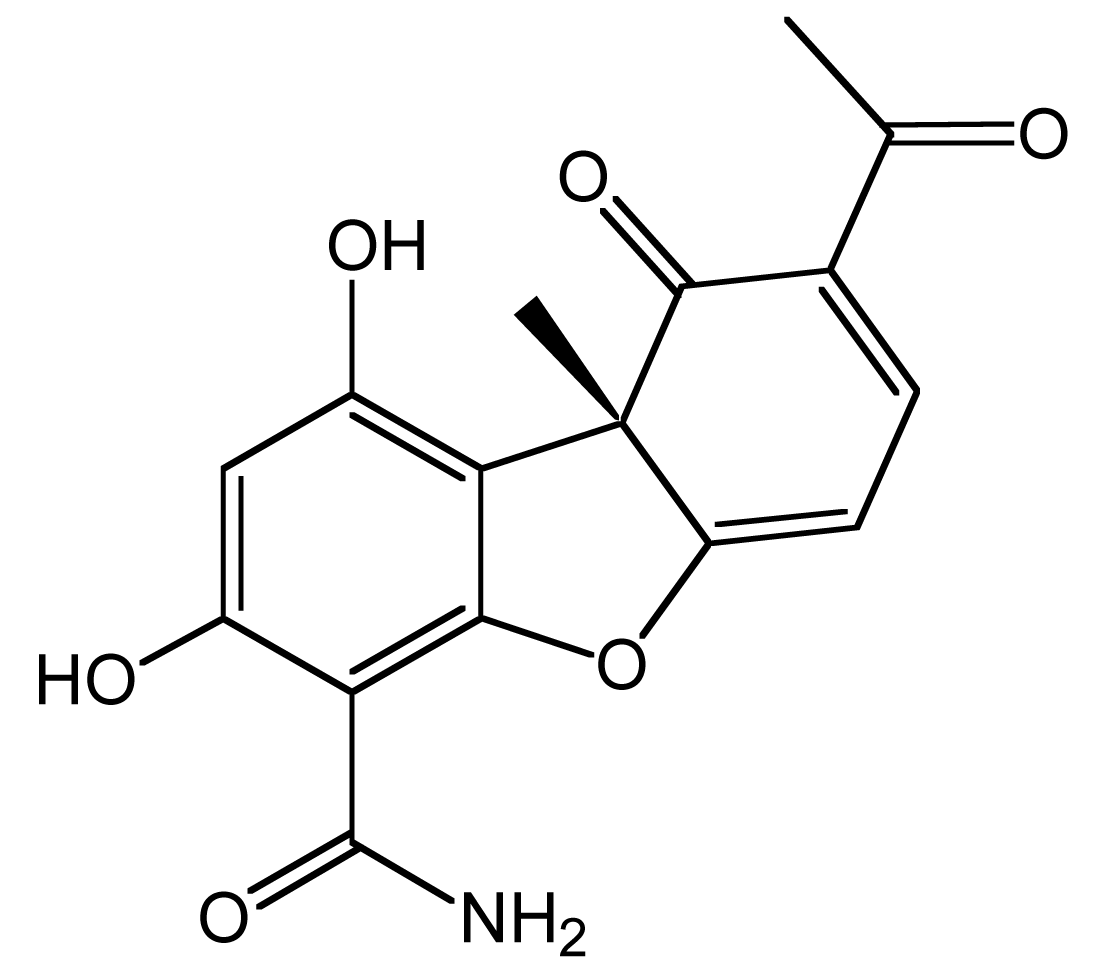

Supplement: Figure S9 — Structure of cercosporamide. The structure of cercoposamide was made using ChemDraw Pro (CyberChem, Inc.). (0.05 MB TIF) [file ppat.1001069.s009.tif]
