# Supplementary material for: PKC Signaling Regulates Drug Resistance of the Fungal Pathogen Candida albicans via Circuitry Comprised of Mkc1, Calcineurin, and Hsp90
Source: PLoS Pathog. 2010 Aug 26;6(8):e1001069. doi: 10.1371/journal.ppat.1001069 (PMC2928802; doi:10.1371/journal.ppat.1001069)
Supplement: Table S1 — Strains used in this study. (0.17 MB DOC) [file ppat.1001069.s010.doc]

**Table S1. Strains used in this study.**

_______________________________________________________________________

Strain Name Genotype Source

_______________________________________________________________________

CaLC79 (CaCi-2) Prototrophic [1,2,3]

CaLC80 (CaCi-3) Prototrophic [1,2,3]

CaLC81 (CaCi-4) Prototrophic [1,2,3]

CaLC82 (CaCi-5) Prototrophic [1,2,3]

CaLC84 (CaCi-7) Prototrophic [1,2,3]

CaLC85 (CaCi-8) Prototrophic [1,2,3]

CaLC86 (CaCi-9) Prototrophic [1,2,3]

CaLC87 (CaCi-11) Prototrophic [1,2,3]

CaLC88 (CaCi-12) Prototrophic [1,2,3]

CaLC89 (CaCi-15) Prototrophic [1,2,3]

CaLC90 (CaCi-16) Prototrophic [1,2,3]

CaLC91 (CaCi-17) Prototrophic [1,2,3]

CaLC155 (SC5314) Prototrophic [4]

CaLC173 (L1, 2307) Prototrophic [5]

CaLC174 (E1, 412) Prototrophic [5]

CaLC175 (E2, 3107) Prototrophic [5]

CaLC176 (L2, 3281) Prototrophic [5]

CaLC177 (E3, 5052) Prototrophic [5]

CaLC178 (L3, 5044) Prototrophic [5]

CaLC179 (E4, 1649) Prototrophic [5]

CaLC180 (L4, 3034) Prototrophic [5]

CaLC181 (E5, 3917) Prototrophic [5]

CaLC182 (L5, 4617) Prototrophic [5]

CaLC239 (SN95) *arg4∆/arg4∆ his1∆/his1∆* [6]

*URA3/ura3::imm434 IRO1/iro1::imm434*

CaLC436 As SN95, *HIS1/his1::TAR-FRT* [7]  *hsp90::CdHIS1/tetO-HSP90*

CaLC648 As SN95, *MKC1/MKC1-6xHIS-FLAG-FRT* This study

*HIS1/his1::TAR-FRT hsp90::CdHIS1/tetO-HSP90*

CaLC660 As SN95, *erg3::FRT/erg3::FRT* [8]

CaLC671 As SN95, *MKC1/mkc1::FRT* This study

CaLC681 As SN95, *MKC1/MKC1-6xHIS-FLAG-FRT*  This study

CaLC700 As SN95, *mkc1::FRT/mkc1::FRT* This study

CaLC720 As SN95, *mkc1::FRT/MKC1-6xHIS-FLAG-FRT* This study

CaLC893 As SN95, *BCK1/bck1::FRT* This study

CaLC896 As SN95, *bck1::FRT/bck1::FRT* This study

CaLC909 As SN95, *cna1::FRT/cna1::FRT* [9]

CaLC948 As SN95, *PKC1/pkc1::FRT CaTAR::HIS3* This study

CaLC1249 As SN95, *MKC1/MKC1-6xHIS-FLAG-FRT*  This study

*HIS1/his1::TAR-FRT hsp90::CdHIS1/tetO-HSP90*

*BCK1/bck1::FRT*

CaLC1255 As SN95, *pkc1::FRT/pkc1::FRT CaTAR::HIS3* This study

CaLC1256 As SN95, *pkc1::FRT::CaPKC1-FRT/pkc1::FRT* This study

*CaTAR::HIS3*

CaLC1258 As SN95, *MKC1/MKC1-6xHIS-FLAG-FRT*  This study

*HIS1/his1::TAR-FRT hsp90::CdHIS1/tetO-HSP90*

*bck1::FRT/bck1::FRT*

CaLC1446 As SC5314, *mid1::frt/mid1::frt* [10]

CaLC1448 As SC5314, *cch1::frt/cch1::frt* [10]

CaLC1449 As SC5314, *mid1::frt/mid1::frt* [10]

*cch1::frt/cch1::frt*

CaLC 1499 *arg4*∆/*arg4*∆ *leu2*∆/*leu2*∆ *his1*∆/*his1*∆ [11]

*URA3/ura3*∆::*imm434 IRO1/iro1*∆::*imm434*

*rlm1∆::LEU2/rlm1∆::HIS1*

CaLC1500 *arg4*∆/*arg4*∆ *leu2*∆/*leu2*∆ *his1*∆/*his1*∆ [11]

*URA3/ura3*∆::*imm434 IRO1/iro1*∆::*imm434*

*rlm1∆::LEU2/rlm1∆::HIS1*

CaLC1501  *arg4*∆/*arg4*∆ *leu2*∆/*leu2*∆ *his1*∆/*his1*∆ [11]

*URA3/ura3*∆::*imm434 IRO1/iro1*∆::*imm434*

*swi4∆::LEU2/swi4∆::HIS1*

CaLC1502  *arg4*∆/*arg4*∆ *leu2*∆/*leu2*∆ *his1*∆/*his1*∆ [11]

*URA3/ura3*∆::*imm434 IRO1/iro1*∆::*imm434*

*swi4∆::LEU2/swi4∆::HIS1*

ScLC3 (D2-400) As S288C, *ura3::KAN*, Pdr1 T817K [12]

ScLC151 (BY4741) *his3Δ leu2Δ met15Δ ura3Δ*  [13]

ScLC10 As BY4741, *erg3::KAN* Deletion Library

ScLC14 As BY4741, *cnb1::KAN* Deletion Library

ScLC511 As BY4741, *pkc1-3::KAN* Charlie Boone

ScLC538 As BY4741, *bck1::KAN* Deletion Library

ScLC541 As BY4741, *slt2::KAN* Deletion Library

ScLC542 As BY4741, *rlm1::KAN* Deletion Library

ScLC1231 As BY4741, *4XCDRE-lacZ-URA3-TRP* This study

ScLC1241 As BY4741, *swi4::KAN* Deletion Library

ScLC1252 As BY4741, *mid1::KAN* Deletion Library

ScLC1253 As BY4741, *cch1::KAN* Deletion Library

ScLC1254 As BY4741, *swi6::KAN* Deletion Library

ScLC1356 As BY4741, *cch1::NAT* This study

ScLC1357 As BY4741, *swi6::NAT* This study

ScLC1359 As BY4741, *slt2::KAN* This study

*4XCDRE-lacZ-URA3-TRP1*

ScLC1360 As BY4741, *cnb1::KAN* This study

*4XCDRE-lacZ-URA3-TRP1*

ScLC 1361 As BY4741, *rlm1::HPH* This study

ScLC1413 As BY4741, *mid1::KAN* *cch1::NAT* This study

ScLC1415 As BY4741, *swi4::KAN rlm1::HPH* This study

ScLC1416 As BY4741, *swi6::NAT rlm1::HPH* This study

ScLC1488 As BY4741, *swi4::KAN* *cch1::NAT* This study

ScLC1489 As BY4741, *swi4::KAN* *cch1::NAT rlm1::HPH* This study

**______________________________________________________________________________**
