# Supplementary material for: PKC Signaling Regulates Drug Resistance of the Fungal Pathogen Candida albicans via Circuitry Comprised of Mkc1, Calcineurin, and Hsp90
Source: PLoS Pathog. 2010 Aug 26;6(8):e1001069. doi: 10.1371/journal.ppat.1001069 (PMC2928802; doi:10.1371/journal.ppat.1001069)
Supplement: Table S2 — Plasmids used in this study (0.03 MB DOC) [file ppat.1001069.s011.doc]

**Table S2. Plasmids used in this study.**

­­­­­­­­­­______________________________________________________________________________

Plasmid Description (Backbone) Source

______________________________________________________________________________

pLC1 (pAG25) natMX4, ampR (pFA6) [12]

pLC3 (pAG32) hphMX4, ampR (pFA6) [12]

pLC49 *FLP-CaNAT*, ampR [13]

pLC74 *CYC1p-4XCDRE-LACZ,* ampR, *TRP1* [14]

pLC362 *CaMKC1*-KO, ampR, NAT (pLC49) This study

pLC363 *CaMKC1-6xHISFLAG*, ampR, NAT (pLC49) This study

pLC442 *CaBCK1*-KO, ampR, NAT (pLC49) This study

pLC470 *CaPKC1-*KO, ampR, NAT (pLC49) This study

pLC522 *CaPKC1,* ampR, NAT (pLC49) This study

______________________________________________________________________________
