# Supplementary material for: PKC Signaling Regulates Drug Resistance of the Fungal Pathogen Candida albicans via Circuitry Comprised of Mkc1, Calcineurin, and Hsp90
Source: PLoS Pathog. 2010 Aug 26;6(8):e1001069. doi: 10.1371/journal.ppat.1001069 (PMC2928802; doi:10.1371/journal.ppat.1001069)
Supplement: Table S3 — Primers used in this study. (0.04 MB DOC) [file ppat.1001069.s012.doc]

**Table S3. Primers used in this study.**

­­­­­­­­­­­­­­Prime Description Sequence

oLC101 KanB CTGCAGCGAGGAGCCGTAAT

oLC103 cnb1-A ATAATTTGTTTCCCTCGACTTCTCT

oLC150 cnb1-D TTTGAGGAAAATCTCTGGTATTCTG

oLC274 pJK863down-F CTGTCAAGGAGGGTATTCTGG

oLC275 pJK863up-R AAAGTCAAAGTTCCAAGGGG

oLC514 HISFLAG+17-F ACGGTGGTGATTATAAAG

oLC515 CaMKC1+3-R-ApaI TTGCGGGCCCCATTATGGAAATTGGTTC

oLC516 CaMKC1-365-F-ApaI TTGCGGGCCCGAAATAGTGTTGTCAAACC

oLC517 CaMKC1+1528-F-SacII TCCCCGCGGTAGTTGAAACACACAACC

oLC518 CaMKC1+1948-R-SacI CCCGAGCTCCGCTCTATGAAATCAGG

oLC519 CaMKC1+1159-F-ApaI TTGCGGGCCCCCAATTGAGGAACAGCAGCG

oLC520 CaMKC1+1527-6HISFLAG-R-ApaI

TTGCGGGCCCTTATTTATCATCATCATCTTTA

TAATCACCACCGTGGTGGTGGTGGTGGTGCT

GGTGGTCGTTACAGTAG

oLC521 CaMKC1-464-F GCCGTTCAAACGTTCACC

oLC522 CaMKC1+2068-R GATCAATCGAAGACAACC

oLC523 CaMKC1+1034-F CTAAAGAAGAAATTGAGTGCC

oLC761 CaBCK1-573-F CAAATCACCTATACTATCATTG

oLC762 CaBCK1+4687-R CAGAGAGAGAGAGAGATAG

oLC768 ScBCK1+261-F GACCACATCTCAGCTGGAACGC

oLC769 ScBCK1+971-R GTGCGGCCGAGCCGGTATAC

oLC770 ScSLT2+289-F CCAGACGGGTCTATCAATGG

oLC771 ScSLT2+944-R GCTCCAGGGCCTCATCCACGG

oLC886 CaBCK1+1294-F GGAGTCACCTGAAGCTGGTGG

oLC887 CaBCK1+2396-R CTTGATGTTGAGTGGGGAGATATTTTGG

oLC946 CaPKC1-624-F-ApaI TTGCGGGCCCGGTATTCTGAGAGTTTAG

oLC947 CaPKC1+3-R-ApaI TTGCGGGCCCCATATTGGAATTACACTG

oLC948 CaPKC1+3296-F-NotI ATAAGAATGCGGCCGCCTGACAATGCAACG ATTTGATT

oLC949 CaPKC1+3732-R-SacII GGCACCGCGGCAAACTACCTGCCACAGCGG

oLC950 CaPKC1-706-F CCAGATTGCAATTGTCG

oLC951 CaPKC1+3880-R GAGAGCATTGCCAACGTGGCTG

oLC954 CaPKC1+0-F-NotI ATAAGAATGCGGCCGCATGTCGACGTCACA

GCCG

oLC956 CaPKC1+439-R CCATAAGATAAAGATGAG

oLC958 E.coli B-gal + 476-R GCCATCAAAAATAATTCGCG

oLC959 E.coli B-gal + 123-F GCAGCACATCCCCCTTTCGC

oLC1027 CaPKC1-429-F-ApaI TTGCGGGCCCCCTACTTTTCGCCGTACTCT

AAG

oLC1028 CaPKC1+3713-R-ApaI TTGCGGGCCCGTCACTACAAGAACTGATACCCG

oLC1029 CaPKC1+3714-F-NotI ATAAGAATGCGGCCGCCGCTGTGGCAG

GTAGTTTGAAG

oLC1030 CaPKC1+4119-R-SacII TCCCCGCGGCCATTGTCTTGGTGTATGCC

oLC1042 CaPKC1+3200-F CACCAAGATTGACACCAGTGG

oLC1138 MX-Fwd ACATGGAGGCCCAGAATACC

oLC1139 MX-Rev CAGTATAGCGACCAGCATTC

oLC1180 ScRLM1+681-F GCCTTCATCCAGTTCATCTTCTC

oLC1181 ScRLM1+1393-R GCTGGGTTTGTCTTTGAGC

oLC1182 ScSWI4+551-F CGGCTACAACTACAGCTGCG

oLC1183 ScSWI4+1256-R CAGTACTCATTATGGTGATC

oLC1184 ScSWI6+292-F GGTTTGTTCCAGGATGCATTCG

oLC1185 ScSWI6+967-R GTAATGGTGTGTTTCCATGCTC

oLC1186 ScMID1+321-F CCAAATGCCCATGAATAGATC

oLC1187 ScMID1+1044-R GCCGCCCACACTTGACAACCC

oLC1188 ScCCH1+337-F GCCGCTGAATCTTCCCGGCG

oLC1189 ScCCH1+1041-R ACGAATACCGCCTATGCAAC

oLC1190 ScCNB1+248-F GCTGGACGTATAATGGAGG

oLC1191 ScCNB1+855-R CACCCCGCAGTTATTACATG

oLC752 CaGPD1+570-F AGTATGTGGAGCTTTACTGGGA

oLC753 CaGPD1+766-R CAGAAACACCAGCAACATCTTC

oLC754 CaHSP90+832-F CCATCTGATATCACTCAAGATG

oLC755 CaHSP90+1040-R AGTGATAAACACTCTACGGACG

oLC1015 ScACT1-F GTTTAGAGGTTGCTGCTTTG

oLC1016 ScACT1-R TGGTGACAATACCGTGTTC

oLC1286 ScCNA1+388-F CTGTTTGAAGTTGGCGGTGACC

oLC1287 ScCNA1+560-R GAGGTCAAGTGCTTACACTCATGG

oLC1288 ScCNA2+475-F GGCGGAGATCCGGCCACTACATC

oLC1289 ScCNA2+608-R CCCCTCAGTAGCCAGAAATGGTCG

oLC1290 ScCNB1+151-F GGCGTTTCGTCAAACCCTCTTGC

oLC1291 ScCNB1+324-F CCGTCCTTGTCAATGTCGTAG

oLC1292 CaCNB1+472-F GCAGTCAACACCGATACAATTGCC

oLC1293 CaCNB1+614-R GGTGTTACAGCAGGTATTGGCC

oLC1294 CaCNA1+109-F CAACGACAAATCTTGCAAAATCCC

oLC1295 CaCNA1+410-R CATATAGTTACTGGTGCCGGAACAC

oLC1328 ScCRZ1+261-F CGTGAACAGCTTGCTGTCTC

oLC1329 ScCRZ1+466-R CGTCCCACTGAAATGTATCAG

oLC1330 CaCRZ1+435-F GCCTCAGTTTACCACTAACG

oLC1331 CaCRZ1+665-R GGAGATGCAGGTTGTGATG

oLC1332 CaMKC1+109-F GGTGCCTATGGTATTGTGTG

oLC1333 CaMKC1+301-R CCCTGAAGAACTGCAACAAC

oLC1432 CaFGR22(PLC3)+560-F CCACCAACGATGACCGTGGTC

oLC1433 CaFGR22(PLC3)+766-R CAGCAATAGGTTGTGGCCAG

oLC1434 CaUTR2+136-F GGTATCTGTGGTACTGGGGC

oLC1435 CaUTR2+394-R CAACGGTACCAGTGGTATG

______________________________________________________________________________
