# Supplementary material for: PKC Signaling Regulates Drug Resistance of the Fungal Pathogen Candida albicans via Circuitry Comprised of Mkc1, Calcineurin, and Hsp90
Source: PLoS Pathog. 2010 Aug 26;6(8):e1001069. doi: 10.1371/journal.ppat.1001069 (PMC2928802; doi:10.1371/journal.ppat.1001069)
Supplement: Text S1 — Supporting Materials and Methods. (0.06 MB DOC) [file ppat.1001069.s013.doc]

**Text S1**

**Supporting Materials and Methods**

***Strain Construction***

**CaLC648:** The plasmid pLC363 was digested with BssHII to liberate the cassette to C-terminally tag with 6xHis-FLAG and was transformed in CaLC436. For NAT resistant transformants, proper integration was verified by PCR using primers oLC523/275 (681bp) and oLC522/274 (682bp). The *SAP2* promoter was induced to drive expression of FLP recombinase to excise the NAT marker cassette. The presence of the 6xHis-FLAG tag in the genome after flipping was verified using oLC514/518 (640bp).

**CaLC671:** The plasmid pLC362 was digested with BssHII to liberate the *MKC1* knock out cassette and was transformed into CaLC239. For NAT resistant transformants, proper integration was verified by PCR using primers for proper integration with oLC521/275 (604bp) and oLC522/274 (682bp). The *SAP2* promoter was induced to drive expression of FLP recombinase to excise the NAT marker cassette. Presence of the knocked out allele after flipping was confirmed with oLC521/522 (1.1kb).

**CaLC681:** The plasmid pLC363 was digested with BssHII to liberate the cassette to C-terminally tag with 6xHis-FLAG and was transformed in CaLC239. For NAT resistant transformants, proper integration was verified by PCR using primers oLC523/275 (681bp) and oLC522/274 (682bp). The *SAP2* promoter was induced to drive expression of FLP recombinase to excise the NAT marker cassette. The presence of the 6xHis-FLAG tag in the genome after flipping was verified using oLC514/518 (640bp).

**CaLC700:** The plasmid pLC362 was digested with BssHII to liberate the *MKC1* knock out cassette and was transformed into CaLC671. For NAT resistant transformants, proper integration was verified by PCR using primers for proper integration with oLC521/275 (604bp) and oLC522/274 (682bp). The *SAP2* promoter was induced to drive expression of FLP recombinase to excise the NAT marker cassette. Absence of a wild-type *MKC1* allele in the *mkc1* deletion mutant was confirmed by the absence of a band when PCR was performed using primers oLC518/519 (absence of 789bp band).

**CaLC720:** The plasmid pLC362 was digested with BssHII to liberate the *MKC1* knock out cassette and was transformed into CaLC681. For NAT resistant transformants, proper integration was verified by PCR using primers for proper integration with oLC521/275 (604bp) and oLC522/274 (682bp). To ensure that the knock-out construct had not integrated in the 6xHis-FLAG tagged MKC1 allele, the presence of the 6xHis-FLAG tag in the genome was verified using oLC514/518 (640bp). The *SAP2* promoter was induced to drive expression of FLP recombinase to excise the NAT marker cassette. Presence of the knocked out allele after flipping was confirmed with oLC521/522 (1.1kb).

**CaLC893:** The plasmid pLC442 was digested with KpnI and SacI to liberate the *BCK1* knock out cassette and was transformed into CaLC239. For NAT resistant transformants, proper integration was verified by PCR using primers for proper integration with oLC761/275 (713bp) and oLC762/274 (884bp). The *SAP2* promoter was induced to drive expression of FLP recombinase to excise the NAT marker cassette. Presence of the knocked out allele after flipping was confirmed with oLC761/762 (1.2kb).

**CaLC896:** The plasmid pLC442 was digested with KpnI and SacI to liberate the *BCK1* knock out cassette and was transformed into CaLC893. For NAT resistant transformants, proper integration was verified by PCR using primers for proper integration with oLC761/275 (713bp) and oLC762/274 (884bp). The *SAP2* promoter was induced to drive expression of FLP recombinase to excise the NAT marker cassette. Absence of a WT *BCK1* allele in the *bck1* deletion mutant was confirmed by the absence of a band when PCR was performed using primers oLC886/887 (absence of 1.1kb band)

**CaLC948:** The plasmid pLC470 was digested with BssHII to liberate the *PKC1* knock out cassette and was transformed into CaLC206. For NAT resistant transformants, proper integration was verified by PCR using primers oLC950/275 (839bp) and oLC951/274 (738bp). The *SAP2* promoter was induced to drive expression of FLP recombinase to excise the NAT marker cassette. Presence of the knocked out allele after flipping was confirmed with oLC950/951 (1.4kb).

**CaLC1249:** The plasmid pLC442 was digested with KpnI and SacI to liberate the *BCK1* knock out cassette and was transformed into CaLC648. For NAT resistant transformants, proper integration was verified by PCR using primers for proper integration with oLC761/275 (713bp) and oLC762/274 (884bp). The *SAP2* promoter was induced to drive expression of FLP recombinase to excise the NAT marker cassette. Presence of the knocked out allele after flipping was confirmed with oLC761/762 (1.2kb).

**CaLC1255:** The plasmid pLC470 was digested with BssHII to liberate the *PKC1* knock out cassette and was transformed into CaLC948. For NAT resistant transformants, proper integration was verified by PCR using primers for proper integration with oLC950/275 (839bp) and oLC951/274 (738bp). The *SAP2* promoter was induced to drive expression of FLP recombinase to excise the NAT marker cassette. Absence of a wild-type *PKC1* allele in the *pkc1* deletion mutant was confirmed by the absence of a band when PCR was performed using primers oLC954/956 (absence of 439bp band).

**CaLC1256:** The plasmid pLC522 was digested with BssHII to liberate the *PKC1* ORF complementation construct and was transformed into CaLC1255. For NAT resistant transformants, proper integration was verified by PCR using primers for proper integration with oLC1042/275 (660bp) and oLC1030/274 (555bp). The *SAP2* promoter was induced to drive expression of FLP recombinase to excise the NAT marker cassette. Presence of the wild-type *PKC1* allele was verified using oLC954/956 (439bp).

**CaLC1258:** The plasmid pLC442 was digested with KpnI and SacI to liberate the *BCK1* knock out cassette and was transformed into CaLC1249. For NAT resistant transformants, proper integration was verified by PCR using primers for proper integration with oLC761/275 (713bp) and oLC762/274 (884bp). The *SAP2* promoter was induced to drive expression of FLP recombinase to excise the NAT marker cassette. Absence of a wild-type *BCK1* allele in the *bck1* deletion mutant was confirmed by the absence of a band when PCR was performed using primers oLC886/887 (absence of 1.1kb band).

**ScLC1231:** To knock out *TRP1* in the BY4741 wild-type strain the hygromycin B resistance gene was amplified from pLC3 using primers with homology to the *TRP1* locus, oLC868/oLC869 (2kb). The PCR product was transformed into ScLC151. Hygromycin B resistant transformants were tested for deletion of *TRP1* by replica plating onto SD plates with and without tryptophan. The tryptophan auxotroph ScLC151 was then transformed with pLC74 digested with StuI. This allows for integration at *URA3* and selection for tryptophan prototrophy. Presence of *E. coli lacZ* was confirmed using oLC958/C959 (353bp).

**ScLC1356:** The NATMX4 resistance cassette was amplified from pLC1 using oLC1138/1139 (1.1kb). The PCR product was transformed into ScLC1253 (*cch1::KAN).* NAT resistant and G418 sensitive transformants were selected to ensure the NATMX4 cassette had replaced the KANMX cassette. The absence of wild-type *CCH1* was verified by PCR using oLC1188/1189 (absence of a 704bp band)

**ScLC1357:** The NATMX4 resistance cassette was amplified from pLC1 using oLC1138/1139 (1.1kb). The PCR product was transformed into ScLC1254 (*swi6::KAN).* NAT resistant and G418 sensitive transformants were selected to ensure the NATMX4 cassette had replaced the KANMX cassette. The absence of wild-type *SWI6* was verified by PCR using oLC1184/1185 (absence of 675bp band)

**ScLC1359:** Made by mating ScLC1231 with a MATα *slt2:KAN*. Diploids were sporulated in liquid medium and tetrads were dissected. Meiotic progeny were tested for *slt2Δ* by PCR using oLC612/101 (609bp) and the presence of *E. coli* *lacZ* was confirmed using oLC958/959 (353bp). Absence of WT *SLT2* in the genome was verified by PCR using oLC770/771 (absence of a 655bp band).

**ScLC1360:** Made by transforming ScLC1231 with a *cnb1::*KANMX PCR product that was amplified from ScLC14 (BY4741, *cnb1Δ)* using oLC103/150 (2.2kb). For KAN resistant transformants, proper integration of the *CNB1* knock out construct was verified by PCR using oLC101/103(603bp) and absence of wild-type *CNB1* was a determined using oLC1190/1191(absence of a 607bp band).

**ScLC1361:** The HPHMX4 resistance cassette was amplified from pLC3 using oLC1138/1139 (1.6kb). The PCR product was transformed into ScLC542 (*rlm1::KAN).* Hygromycin B resistant and G418 sensitive transformants were selected to ensure the HPHMX4 cassette had replaced the KANMX cassette. The absence of wild-type *RLM1* was verified by PCR using oLC1180/1181 (absence of 712bp band)

***Plasmid Construction***

**pLC362:** A DNA region, approximately 500 base pairs long, that was homologous to a region of DNA immediately downstream of the *MKC1* stop codon was amplified from SC5314 genomic DNA using oLC517 and oLC518 and cloned into pLC49 at SacII and SacI. A DNA region, approximately 500 base pairs long, that was homologous to a region of DNA immediately upstream of *MKC1* start codon was amplified from SC5314 genomic DNA with primers oLC515 and oLC516 and cloned into pLC49 containing the downstream homology at ApaI. The presence of the inserts within the vector was tested by PCR with oLC274/oLC518 and oLC275/oLC516. The cassette to knock out *MKC1* can be excised with BssHII.

**pLC363:** A DNA region that is homologous to the C-terminal part of *MKC1,* immediately before the stop codon, was amplified from SC5314 genomic DNA using primers oLC519 and oLC520, which contains the 6xHis-FLAG tag within the primer. This product was cloned into pLC49 at ApaI and proper integration was verified by PCR using primers oLC519 and oLC275. A DNA region that is homologous to a fragment of DNA downstream of the coding region of *MKC1*was amplified from SC5314 genomic DNA using primers oLC517 and oLC518 and cloned into pLC49 containing the upstream region of homology and the 6xHis-FLAG tag at SacII and SacI. Proper integration was verified by PCR using primers oLC518 and oLC274. The cassette to C-terminally 6xHis-FLAG tag *MKC1* can be excised with BssHII.

**pLC442:** A DNA region, approximately 500 base pairs long, that was homologous to a region of DNA immediately downstream of the *BCK1* stop codon was amplified from SC5314 genomic DNA using oLC765 and oLC766 and cloned into pLC49 at SacII and SacI. A DNA region, approximately 500 base pairs long, that was homologous to a region of DNA immediately upstream of the *BCK1* start codon was amplified from SC5314 genomic DNA with primers oLC763 and oLC764 and cloned into pLC49 containing the downstream homology at KpnI and ApaI. The presence of the inserts within the vector was tested by PCR with oLC274/oLC766 and oLC275/oLC763. The cassette to knock out *BCK1* can be excised with KpnI and SacI.

**pLC470:** A DNA region, approximately 500 base pairs long, that was homologous to a region of DNA immediately upstream of the *PKC1* start codon was amplified from SC5314 genomic DNA using oLC946 and oLC947 and cloned into pLC49 at ApaI. A DNA region, approximately 500 base pairs long, that was homologous to a region of DNA immediately downstream of the *PKC1* stop codon was amplified from SC5314 genomic DNA with primers oLC948 and oLC949 and cloned into pLC49 containing the upstream homology at NotI and SacII. The presence of the inserts within the vector was tested by PCR with oLC274/oLC949 and oLC275/oLC946. The cassette to knock out *BCK1* can be excised with KpnI and SacI.

**pLC522:** A DNA region that was homologous to a region of DNA corresponding to the entire *PKC1* ORF and some of the *PKC1* promoter and terminator regions was amplified from SC5314 genomic DNA with primers oLC1027 and oLC1028 and was cloned into pLC49 at ApaI. A DNA region that was homologous to a region of DNA further downstream in the *PKC1* terminator region was amplified from SC5314 genomic DNA with primers oLC1029 and oLC1030 and was cloned into pLC49 containing *HSP90* at NotI and SacII. Proper integration was verified by PCR using primers oLC274/oLC1030 and oLC275/oLC1042. The *PKC1* complementation cassette can be liberated by digestion with BssHII.

**Supplemental References**

1. White TC (1997) Increased mRNA levels of ERG16, CDR, and MDR1 correlate with increases in azole resistance in Candida albicans isolates from a patient infected with human immunodeficiency virus. Antimicrob Agents Chemother 41: 1482-1487.

2. White TC (1997) The presence of an R467K amino acid substitution and loss of allelic variation correlate with an azole-resistant lanosterol 14alpha demethylase in Candida albicans. Antimicrob Agents Chemother 41: 1488-1494.

3. White TC, Pfaller MA, Rinaldi MG, Smith J, Redding SW (1997) Stable azole drug resistance associated with a substrain of Candida albicans from an HIV-infected patient. Oral Dis 3 Suppl 1: S102-109.

4. Jones T, Federspiel NA, Chibana H, Dungan J, Kalman S, et al. (2004) The diploid genome sequence of Candida albicans. Proc Natl Acad Sci U S A 101: 7329-7334.

5. Perea S, Lopez-Ribot JL, Kirkpatrick WR, McAtee RK, Santillan RA, et al. (2001) Prevalence of molecular mechanisms of resistance to azole antifungal agents in Candida albicans strains displaying high-level fluconazole resistance isolated from human immunodeficiency virus-infected patients. Antimicrob Agents Chemother 45: 2676-2684.

6. Noble SM, Johnson AD (2005) Strains and strategies for large-scale gene deletion studies of the diploid human fungal pathogen Candida albicans. Eukaryot Cell 4: 298-309.

7. Shapiro RS, Uppuluri P, Zaas AK, Collins C, Senn H, et al. (2009) Hsp90 Orchestrates Temperature-Dependent Candida albicans Morphogenesis via Ras1-PKA Signaling. Curr Biol.

8. Robbins N, Collins C, Morhayim J, Cowen LE (2010) Metabolic control of antifungal drug resistance. Fungal Genet Biol 47: 81-93.

9. Singh SD, Robbins N, Zaas AK, Schell WA, Perfect JR, et al. (2009) Hsp90 Governs Echinocadin Resistance in the Pathogenic Yeast Candida albicans via Calcineurin. PLoS Pathog In Press.

10. Reedy JL, Filler SG, Heitman J (2009) Elucidating the Candida albicans calcineurin signaling cascade controlling stress response and virulence. Fungal Genet Biol.

11. Homann OR, Dea J, Noble SM, Johnson AD (2009) A phenotypic profile of the Candida albicans regulatory network. PLoS Genet 5: e1000783.

12. Anderson JB, Sirjusingh C, Parsons AB, Boone C, Wickens C, et al. (2003) Mode of selection and experimental evolution of antifungal drug resistance in Saccharomyces cerevisiae. Genetics 163: 1287-1298.

13. Giaever G, Chu AM, Ni L, Connelly C, Riles L, et al. (2002) Functional profiling of the Saccharomyces cerevisiae genome. Nature 418: 387-391.

14. Goldstein AL, McCusker JH (1999) Three new dominant drug resistance cassettes for gene disruption in Saccharomyces cerevisiae. Yeast 15: 1541-1553.

15. Shen J, Guo W, Kohler JR (2005) CaNAT1, a heterologous dominant selectable marker for transformation of Candida albicans and other pathogenic Candida species. Infect Immun 73: 1239-1242.

16. Stathopoulos AM, Cyert MS (1997) Calcineurin acts through the CRZ1/TCN1-encoded transcription factor to regulate gene expression in yeast. Genes Dev 11: 3432-3444.
